# Comparative RNA-seq analysis of resistant and susceptible banana genotypes reveals molecular mechanisms in response to *banana bunchy top virus* (BBTV) infection

**DOI:** 10.1038/s41598-023-45937-z

**Published:** 2023-10-31

**Authors:** Darlon V. Lantican, Jen Daine L. Nocum, Anand Noel C. Manohar, Jay-Vee S. Mendoza, Roanne R. Gardoce, Grace C. Lachica, Lavernee S. Gueco, Fe M. Dela Cueva

**Affiliations:** 1 Institute of Plant Breeding, College of Agriculture and Food Science, University of the Philippines Los Baños, College, 4031 Laguna, Philippines; 2 Philippine Genome Center – Program for Agriculture, Livestock, Fisheries, Forestry, Office of the Vice Chancellor for Research and Extension, University of the Philippines Los Baños, College, 4031 Laguna, Philippines

**Keywords:** Transcriptomics, Gene expression, Gene regulation, Sequencing, Plant molecular biology, Biotic

## Abstract

Bananas hold significant economic importance as an agricultural commodity, serving as a primary livelihood source, a favorite fruit, and a staple crop in various regions across the world. However, Banana bunchy top disease (BBTD), which is caused by *banana bunchy top virus* (BBTV), poses a considerable threat to banana cultivation. To understand the resistance mechanism and the interplay of host suitability factors in the presence of BBTV, we conducted RNA-seq-based comparative transcriptomics analysis on mock-inoculated and BBTV-inoculated samples from resistant (wild *Musa balbisiana*) and susceptible (*Musa acuminata* ‘Lakatan’) genotypes. We observed common patterns of expression for 62 differentially expressed genes (DEGs) in both genotypes, which represent the typical defense response of bananas to BBTV. Furthermore, we identified 99 DEGs exclusive to the 'Lakatan' banana cultivar, offering insights into the host factors and susceptibility mechanisms that facilitate successful BBTV infection. In parallel, we identified 151 DEGs unique to the wild *M. balbisiana*, shedding light on the multifaceted mechanisms of BBTV resistance, involving processes such as secondary metabolite biosynthesis, cell wall modification, and pathogen perception. Notably, our validation efforts via RT-qPCR confirmed the up-regulation of the glucuronoxylan 4-O-methyltransferase gene (14.28 fold-change increase), implicated in xylan modification and degradation. Furthermore, our experiments highlighted the potential recruitment of host's substrate adaptor ADO (30.31 fold-change increase) by BBTV, which may play a role in enhancing banana susceptibility to the viral pathogen. The DEGs identified in this work can be used as basis in designing associated gene markers for the precise integration of resistance genes in marker-assisted breeding programs. Furthermore, the findings can be applied to develop genome-edited banana cultivars targeting the resistance and susceptibility genes, thus developing novel cultivars that are resilient to important diseases.

## Introduction

Banana is a major fruit crop in the Philippines and remains to be a large contributor to the country’s dollar reserve, with an estimated total production of 2.40 million metric tons during the last quarter of 2020^1^. ‘Cavendish’, ‘Saba’ and ‘Lakatan’ varieties had the highest production of 50.4%, 28.5% and 10.7%, respectively. Among the 57 banana cultivars in the Philippines, the most planted varieties are ‘Saba’, ‘Latundan’, ‘Lakatan’, ‘Bungulan’ and ‘Cavendish’. The ‘Cavendish’ variety contributes to about 50% of the country’s total export, being highly preferred by the global market^2^. Nevertheless, the Philippines is still ranked 3rd next to India and China in terms of 2010–2015 average global production but ranked 2nd (next to Ecuador) in terms of 2013 agricultural export value^3^. On a global scale, it's impossible to understate the importance of bananas as a significant agricultural commodity. They serve as a primary source of livelihood and a staple crop in various parts of the world. Consequently, it is imperative to prioritize the safeguarding of the global banana industry against threats that could potentially harm the entire sector. However, the maximum potential in banana production is currently being hindered by biotic and abiotic factors, such as the spread of destructive plant diseases like the banana bunchy top disease (BBTD). Almost all regions cultivating bananas are affected by the prevalence of banana bunchy top disease. This underscores the need to develop next-generation methods for integrating into the global management of the BBTD, such as biotechnology applications.

BBTD, caused by *banana bunchy top virus (*BBTV), is transmitted by an aphid vector (*Pentalonia nigronervosa* Coquerel) and results to stunted growth, and an inability to produce fruits in bananas^4, 5^. The infection also occurs in other banana planting materials, hence the faster spread of the disease^4^. One effective way to control BBTD is early detection of the vector and/or host infection and immediate replacement of infected plantlets with BBTV-free planting materials. Still, deployment of BBTV-resistant banana varieties in conjunction with a high-throughput, rapid and sensitive BBTV detection system is still the most sustainable approach to attaining optimum banana production. BBTV-resistant ‘Mapilak’ banana cultivar was previously developed through gamma irradiation and in vitro induced mutations of *Musa acuminata* ‘Lakatan’^6^. However, the resistance reaction of this cultivar only accounted for 20% of the disease incidence reduction^7^. Hence, identification of new sources of resistance alleles to be introgressed in the existing elite varieties is still an area of further research. Fortunately, the Philippines has local access to natural genetic resources such as wild *Musa balbisiana* accessions with complete resistance against the pathogen^8, 9^.

Comparative RNA-seq analysis has aided in unraveling the mechanisms of disease resistance and identification of host-factor genes in several crops. The response mechanisms of banana during infection of race 1 and tropical race 4 of *Fusarium oxysporum f. sp. cubense*^10^ and *Xanthomonas campestris* pv. *musacearum*^11^ were also effectively elucidated based on this approach. To date, there are still no publications or reports explaining the molecular mechanisms of banana-BBTV interaction with varying resistance and susceptibility responses.

With the availability of confirmed BBTV-resistant wild *M. balbisiana* genotype from the Philippines, identification of host-factors and resistance genes through transcriptomics studies (i.e. RNA-seq) is now possible. Here, we provide insights into the molecular basis of disease resistance and host susceptibility between the wild *M. balbisiana* and BBTV-susceptible genotype ‘Lakatan’.

## Results

### Transcriptomic profiles of resistant and susceptible banana genotypes

Two banana genotypes, wild *Musa balbisiana* (BBTV-resistant^9^) and *Musa acuminata* ‘Lakatan’ (BBTV-susceptible^6^), were mock- and BBTV-inoculated by *Pentalonia nigronervosa* and the RNA samples from young leaf tissues were isolated at 72 h post-inoculation (hpi). In total, 12 RNA samples were sent for next-generation sequencing using Illumina NextSeq 500/550 to represent the total transcriptomic profiles of BBTV-free and BBTV-inoculated resistant and susceptible banana genotypes. Approximately 40–67 million raw reads (75-bp length per read) were generated from each sequencing library. All the raw reads generated from this study were uploaded to NCBI under BioProject Accession number PRJNA746416.

The *M. acuminata* version 2 genome assembly^12^ from the Banana Genome Hub (https://banana-genome-hub.southgreen.fr) was used as reference in the genome-guided mapping of the RNA-seq reads for subsequent differential gene expression transcriptomic analysis. Around 5–14 million trimmed paired-end reads (~ 150-bp length) of the RNA-seq data were observed to uniquely map to the reference genome. On average, 94.56% (mock-inoculated ‘Lakatan’), 94.25% (BBTV-inoculated ‘Lakatan’), 94.32% (mock-inoculated wild *M. balbisiana*), and 94.25% (BBTV-inoculated wild *M. balbisiana*) were mapped to the genome (Supplementary Table S1).

### Changes in expression patterns between wild *M. balbisiana* (resistant) and ‘Lakatan’ (susceptible) in response to BBTV

**Figure 1:**
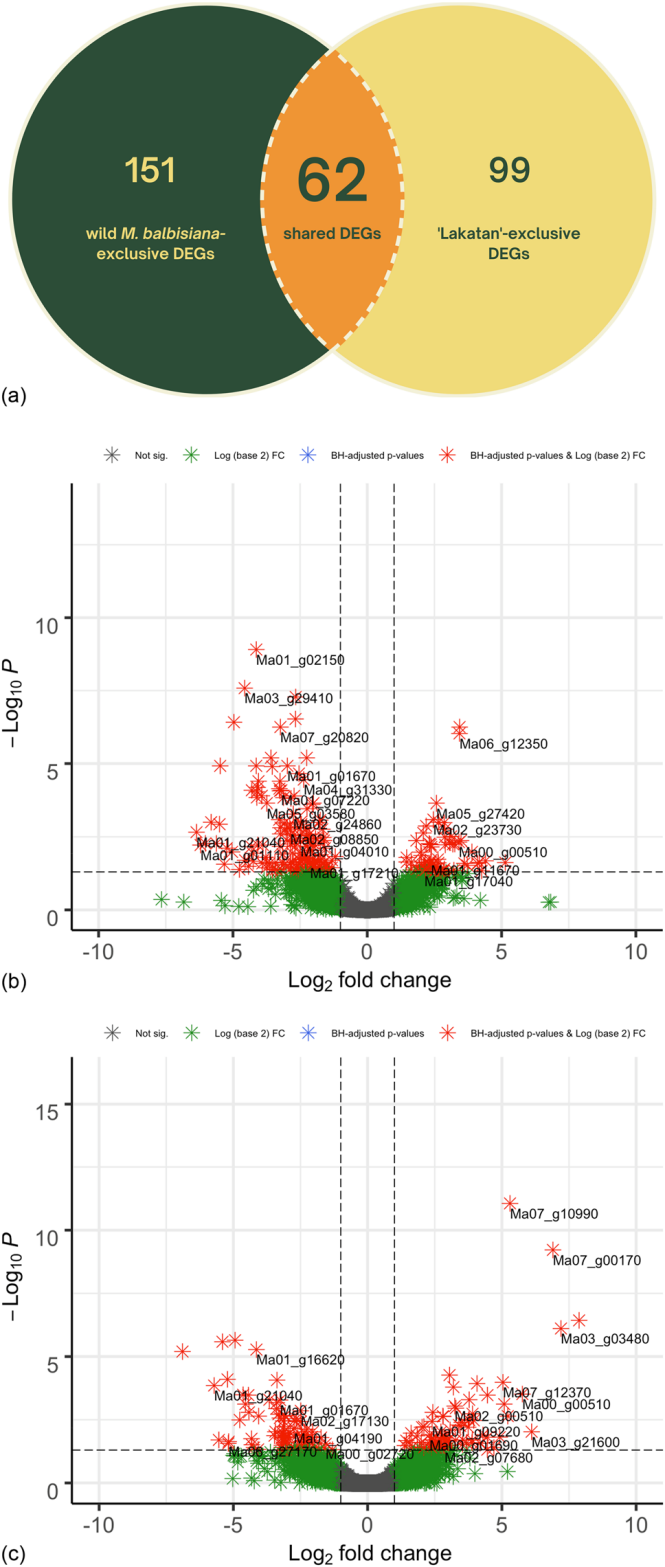
Volcano plots and Venn diagram of DEGs. (**a**) Venn diagram showing the overlap of DEGs between wild *M. balbisiana* and *M. acuminata* ‘Lakatan’. Volcano plots of differentially expressed genes (DEGs) in wild *Musa balbisiana* (**b**) *Musa acuminata* ‘Lakatan’ (**c**) three (3) days post-inoculation. The x-axis shows the fold change difference in the expression of genes, and the y-axis indicates the BH-adjusted *p*-values for the changes in the expression. An absolute value of log2 fold change > 1 and the adjusted *p*-value < 0.05 was set to declare differentially expressed genes (DEGs).

The RSEM-normalized expected count data from BBTV-challenged wild *M. balbisiana* (24,670 genes with non-zero read count) and ‘Lakatan’ (26,901) were statistically compared using the *DESeq2* R package^13^. The DESeq2 pipeline was able to identify 161 and 213 differentially expressed genes based upon BBTV-infection in ‘Lakatan’ and wild *M. balbisiana*, respectively (Supplementary Table S2). A similar trend was also previously observed in the transcriptomic investigation of the response of *M. balbisiana* to *Xanthomonas campestris* pv. *musacearum* causing banana *Xanthomonas* wilt disease*,* in which the resistant *M. balbisiana* had higher number of identified DEGs as compared to the susceptible genotype, Pisang Awak^11^. Among the significantly expressed genes in our study, 77 genes were up-regulated while the other 84 genes were down-regulated in ‘Lakatan’. Meanwhile, the wild *M. balbisiana* was observed to up-regulate 62 genes and down-regulate 151 genes (Volcano plot; Fig. 1). Moreover, comparison of the differentially expressed gene (DEG) profiles between the susceptible and resistant banana accessions revealed 151 DEGs detected only on the wild *M. balbisiana* (Venn Diagram; Fig. 1). Results further showed that the ‘Lakatan’ banana cultivar differentially expressed 99 genes, exclusively. These DEGs might include host factors necessary for a successful BBTV infection in banana. In addition to these DEGs, similar profiles for 62 DEGs were observed on both genotypes, suggesting their possible roles in the recognition and basal defense response mechanism of banana based upon BBTV introduction.

### Shared set of DEGs in resistant and susceptible banana cultivars involved in defense response

Among the identified DEGs, 62 were present in both cultivars in response to BBTV infection (Supplementary Table S2). These include DEGs involved in pathogen response such as cysteine-rich receptor-like kinase 6 (*CRK6*) (Ma06_g23640) and calcium-dependent protein kinase (*CDPK*) (Ma06_g31170) and mitogen-activated protein kinase (*MAPK*)(Ma08_g21950). The gene expression patterns of these genes were the same between wild *M. balbisiana and M. acuminata*, in which *CRK6* and *CDPK* were up-regulated while *MAPK* was down-regulated. CRKs generally plays a role against pathogen attack and programmed cell death^14–17^; specifically, *CRK6* is involved in signaling in response to extracellular reactive oxygen species (ROS)^18^. MAPK, MAPKKK and CDPK regulate plant immune signaling enabling activation of defense genes expression^19, 20^. On the other hand, heat shock proteins (HSPs), chaperone HSP70 (Ma06_g36160) and co-chaperone HSP40/DnaJ (Ma04_g37990), were observed to be both downregulated in the two banana genotypes in response to BBTV. These HSPs generally act as chaperones in pathogenesis, particularly movement, demonstrating their possible role in plant defense or immunity^21^.

Ten differentially expressed transcription factors (TFs) were all observed to be downregulated in both cultivars based upon BBTV introduction that comprise of B-box (BBX) (Ma10_g08680), Dof (Ma11_g14200), six MYB (Ma06_g16330, Ma10_g14160, Ma01_g01670, Ma02_g01300, Ma04_g16680, Ma11_g01360), a C2H2 (Ma03_g14330) and bHLH (Ma08_g10850) TFs. The importance of these transcription factors in defense response of the plant host against pathogen invasion has been previously established in other crops^22–28^.

### Differential expression of genes specific to susceptible cultivar

*M. acuminata*-specific DEGs were further investigated to shed light on the response of a susceptible genotype during BBTV invasion as well as identify potential host factors needed for a successful disease progression. During BBTD development, DEGs involved in the biogenesis of small ribosomal subunit 40S (Ma01_g15210, Ma07_g07360) were up-regulated in the susceptible cultivar. Expression of translation elongation factor *eEF1A* (Ma10_g13420) and eukaryotic translation initiation factor *eIF5A* (Ma11_g09120) were likewise increased by ~ 1.4-fold and ~ 2.6-fold, respectively, most possibly, in response to the invading virus. Being a critical step for successful viral replication, subversion of the host’s protein synthesis machinery and increasing the expression of the components, such as the eEF1A and eIF5A, are needed by the viral pathogen^29, 30^. Genes involved in RNA biosynthesis and processing such as RNA polymerase sigma factor 70 (Ma11_g15590), phosphorolytic exoribonuclease PNP (Ma10_g20770) were also up-regulated in the susceptible variety, signifying their potential involvement in BBTV replication. RNA methyltransferase NOP2 (Ma07_g21640), which is being used by viruses to modify viral RNA and affect host anti-viral systems^31^, was also observed to be upregulated exclusively in ‘Lakatan’ by ~ 2.6-fold.

Interestingly, five HSPs involved in protein homeostasis were differentially expressed in the *M. acuminata* ‘Lakatan’ comprising of HSP70 (Ma02_g18000), HSP60 (Ma11_g20430), Hsp60-co-chaperone Hsp20 (Ma05_g04870), and two homologs of HSP90 (Ma06_g00390; Ma03_g29390). The heat shock proteins available in the host are needed for the virus’ mechanisms for protein processing and have an impact on viral proliferation and counteracting the host’s defense responses^32–34^. Moreover, NAD-dependent glyceraldehyde 3-phosphate dehydrogenase (Ma06_g01470) was upregulated by ~ twofold, previously reported to be implicated in the viral cell-to-cell movement and replication^35^. Hence, the observed upregulation of these sets of DEGs may play a direct role in the development of BBTD in the susceptible banana cultivar.

Notably, two genes integral to the host SCF (SKP1-CUL1-F-box protein) ubiquitin ligase complex, substate adaptor *SKIP35* (Ma03_g02600) and substrate adaptor *ADO* (Ma07_g10990), were both differentially-expressed in BBTV-infected *M. acuminata* with ~ 2.3-fold downregulation and ~ 5.3-fold upregulation, respectively. The SCF ubiquitin ligase complex, which plays a key role in host defense response, has been reported to be hijacked by invading viruses for their own advantage^36^.

### Differential expression of defense-associated genes in resistant cultivar

A total of 151 DEGs were found to be specific in the wild *M. balbisiana.* These will possibly provide insights into the molecular mechanisms of resistance to overcome BBTD disease progression. Among the set of DEGs, six protein kinases were exclusively identified in the resistant genotype, which may play an essential role in pathogen recognition and signaling for a cascade of plant defense mechanisms^37^. Two receptor-like kinase proteins were found to be differently expressed, with RLCK-VI (Ma04_g22180) being upregulated (~ 2.5-fold) and RLCK-VII (Ma04_g09590) being downregulated (~ 3.2-fold). On the other hand, the gene expression of AGC-VIII protein kinase *PHOT2* (Ma08_g26200), LRR-domain containing protein (Ma07_g26720) and serine/threonine kinase (Ma11_g04550), aarF domain-containing protein kinase (Ma04_g25580) in wild *M. balbisiana* were all repressed. Moreover, the gene expression of hypersensitive-induced response protein 1 (Ma07_g25320) in resistant banana cultivar were also observed with increased gene expression by 1.4-fold compared to the mock-inoculated control.

Plant immune system is also regulated by the action of phytohormones, which synergistically and/or antagonistically work in a complex network to respond to the invading pathogen^38, 39^. The genes encoding nematode resistance protein HSPRO2 (Ma10_g05760) and nematode resistance protein-like HSPRO2 (Ma11_g22440), which play roles in hormone-mediated stress signaling^40^, were both downregulated in resistant banana cultivar. Differential gene expression of perception modulator ABAR (Ma02_g08820; downregulated by ~ 2.26-fold), involved in abscisic acid signaling, was also observed in wild *M. balbisiana*. Downregulation of gene expression in auxin transport gene such as auxin efflux carrier component 1a (Ma08_g23810) was also detected, mostly, as a response of BBTV-resistant cultivar to the invading viral pathogen.

Changes in the reduction–oxidation (redox) levels signal defense response mechanisms in pathogen-challenged plant cells^41^. In the BBTV-challenged resistant banana cultivar, a gene implicated in ascorbate-based redox regulation, such as cyt-b561 electron shuttle hemoprotein CYBASC (Ma01_g02150) was downregulated. In response to excessive reactive oxygen species (ROS), as a consequence of pathogen attack, the resistant genotype was observed to increase the expression of a gene encoding for putative protein EARLY RESPONSIVE TO DEHYDRATION 15 (Ma11_g05710), which is a transcription factor attenuating ABA signaling and induces PR genes, leading to the enhanced plant resistance to pathogens^42^.

The resistant response of wild *M. balbisiana* to BBTV was also accompanied by differential expression of genes involved in the synthesis of cell wall modifying enzymes. These resistant genotype-exclusive differentially expressed genes encode glucuronoxylan 4-O-methyltransferase (Ma08_g29650; up-regulated by ~ 3.72-fold) for xylan modification and degradation, and fatty aldehyde dehydrogenase (FADH; Ma08_g30610; up-regulated by ~ 2.1-fold) for cuticular lipid formation. Two genes involved in the biosynthesis of cell wall lignin are all upregulated exclusively in the resistant accession, which include tricetin 3′,4′,5′-O-trimethyltransferase (Ma02_g06390; up-regulated by ~ 2.76), and caffeic acid O-methyltransferase (Ma10_g09050; upregulated by ~ 2.55-fold).

Genes involved in secondary metabolite production, such as terpenoids and phenolics, were also shown to be differentially expressed in the resistant cultivar. Specifically, two genes involved in terpenoid biosynthesis [phytoene synthase (Ma09_g09640), carotenoid beta-ring hydroxylase BCH (Ma11_g19880)] were downregulated while (-)-alpha-terpineol synthase (TPS; Ma04_g08150) was up-regulated. On the other hand, the type-I flavone synthase (FNS; Ma02_g12040) which catalyzes the conversion of flavanones to flavones was downregulated in the BBTV-resistant banana cultivar based upon BBTV infection by ~ 3.4-fold.

### Gene ontology (GO) enrichment of differentially expressed genes

Gene ontology (GO) enrichment analysis (Table 1) was carried out to characterize the gene ontology processes involved in banana during its response to BBTV inoculation and further differentiate the two genotypes based on the uniquely enriched gene ontology terms. Analysis revealed that BP GO terms ‘circadian rhythm’ (GO:0009820), ‘photomorphogenesis’ (GO:0009640), ‘regulation of circadian rhythm’ (GO:0042752), were enriched in both banana genotypes, ‘Lakatan’ and wild *M. balbisiana*.

**Table 1:** Significantly enriched biological process (BP) gene ontology (GO) based on the identified DEGs in BBTV-susceptible and -resistant genotypes.

Genotype	Enrichment comparison	ID	Description	Gene ratio	Bg ratio	p value	p. adjust	q value	Count
Wild *M. balbisiana*	Exclusive	GO:0005985	Sucrose metabolic process	4/184	24/34,320	7.81E−06	0.00115238	0.00095398	4
Wild *M. balbisiana*	Shared	GO:0007623	Circadian rhythm	5/184	87/34,320	0.00010859	0.00678848	0.00561972	5
Wild *M. balbisiana*	Shared	GO:0042752	Regulation of circadian rhythm	4/184	46/34,320	0.00010943	0.00678848	0.00561972	4
Wild *M. balbisiana*	Exclusive	GO:0071456	Cellular response to hypoxia	5/184	116/34,320	0.00041596	0.01873718	0.01551124	5
Wild *M. balbisiana*	Shared	GO:0009640	Photomorphogenesis	4/184	66/34,320	0.00044461	0.01873718	0.01551124	4
Wild *M. balbisiana*	Exclusive	GO:0009704	De-etiolation	2/184	10/34,320	0.00125058	0.03689217	0.03054053	2
Wild *M. balbisiana*	Exclusive	GO:0072488	Ammonium transmembrane transport	2/184	12/34,320	0.00182129	0.04218209	0.03491969	2
Wild *M. balbisiana*	Exclusive	GO:0015977	Carbon fixation	2/184	13/34,320	0.00214485	0.04218209	0.03491969	2
Wild *M. balbisiana*	Exclusive	GO:0010597	Green leaf volatile biosynthetic process	3/184	49/34,320	0.0023301	0.04296127	0.03556473	3
Wild *M. balbisiana*	Exclusive	GO:0009813	Flavonoid biosynthetic process	3/184	51/34,320	0.0026132	0.04534676	0.03753951	3
Lakatan	Shared	GO:0042752	Regulation of circadian rhythm	6/142	46/34,320	3.69E−08	9.84E-06	7.68E-06	6
Lakatan	Shared	GO:0007623	Circadian rhythm	7/142	87/34,320	7.93E−08	1.06E-05	8.26E-06	7
Lakatan	Exclusive	GO:0048511	Rhythmic process	5/142	62/34,320	6.05E−06	0.00040366	0.0003151	5
Lakatan	Shared	GO:0009640	Photomorphogenesis	5/142	66/34,320	8.24E−06	0.0004401	0.00034355	5
Lakatan	Exclusive	GO:0009637	Response to blue light	4/142	34/34,320	1.18E−05	0.00052635	0.00041087	4
Lakatan	Exclusive	GO:0006457	Protein folding	6/142	201/34,320	0.00019791	0.00754897	0.00589275	6
Lakatan	Exclusive	GO:0009408	Response to heat	5/142	156/34,320	0.00049422	0.014662	0.0114452	5
Lakatan	Exclusive	GO:0009615	Response to virus	3/142	36/34,320	0.00044797	0.014662	0.0114452	3
Lakatan	Exclusive	GO:0051085	Chaperone cofactor-dependent protein refolding	3/142	47/34,320	0.0009841	0.01714831	0.01338603	3
Lakatan	Exclusive	GO:0006354	DNA-templated transcription, elongation	2/142	12/34,320	0.00109184	0.01714831	0.01338603	2
Lakatan	Exclusive	GO:0042026	Protein refolding	3/142	44/34,320	0.0008111	0.01714831	0.01338603	3
Lakatan	Exclusive	GO:0010187	Negative regulation of seed germination	2/142	13/34,320	0.00128686	0.0190884	0.01490047	2
Lakatan	Exclusive	GO:0009649	Entrainment of circadian clock	3/142	54/34,320	0.00147392	0.02071243	0.01616819	3
Lakatan	Exclusive	GO:0045892	Negative regulation of transcription, DNA-templated	6/142	307/34,320	0.00180666	0.02297043	0.01793079	6
Lakatan	Exclusive	GO:0009266	Response to temperature stimulus	2/142	15/34,320	0.00172293	0.02297043	0.01793079	2
Lakatan	Exclusive	GO:0006396	RNA processing	4/142	129/34,320	0.00207308	0.02406572	0.01878578	4
Lakatan	Exclusive	GO:2000028	Regulation of photoperiodism, flowering	3/142	76/34,320	0.00390993	0.041758	0.03259643	3
Lakatan	Exclusive	GO:0034620	Cellular response to unfolded protein	2/142	24/34,320	0.00441979	0.04370685	0.0341177	2
Lakatan	Exclusive	GO:0009058	Biosynthetic process	4/142	164/34,320	0.00489616	0.04507847	0.0351884	4
Lakatan	Exclusive	GO:0048586	Regulation of long-day photoperiodism, flowering	2/142	25/34,320	0.00479115	0.04507847	0.0351884	2

The entire list containing the significantly enriched molecular function (MF) GO, and cellular component (CC) GO can be found as Supplementary Table S3.

Various biological processes were exclusively enriched from the transcriptome data of each banana genotype. In ‘Lakatan’, gene ontology terms related to the biological processes such as those involved in ‘cellular response to unfolded protein’ (GO:0034620), protein refolding (GO:0051085; GO:0006457; GO:0042026), regulation of photoperiodism (GO:0048586; GO:2000028) and entrainment of circadian rhythm (GO:0009649) were observed. In contrast, the wild *M. balbisiana* was observed to be enriched on the gene ontology biological processes terms such as ‘sucrose metabolic process’ (GO:0005985), ‘ammonium transmembrane transport’ (GO:0072488), ‘cellular response to hypoxia’ (GO:0071456), ‘green leaf volatile biosynthetic process’ (GO:0010597) and ‘flavonoid biosynthetic process’ (GO:0009813).

### RT-qPCR validation of DEGs

**Figure 2:**
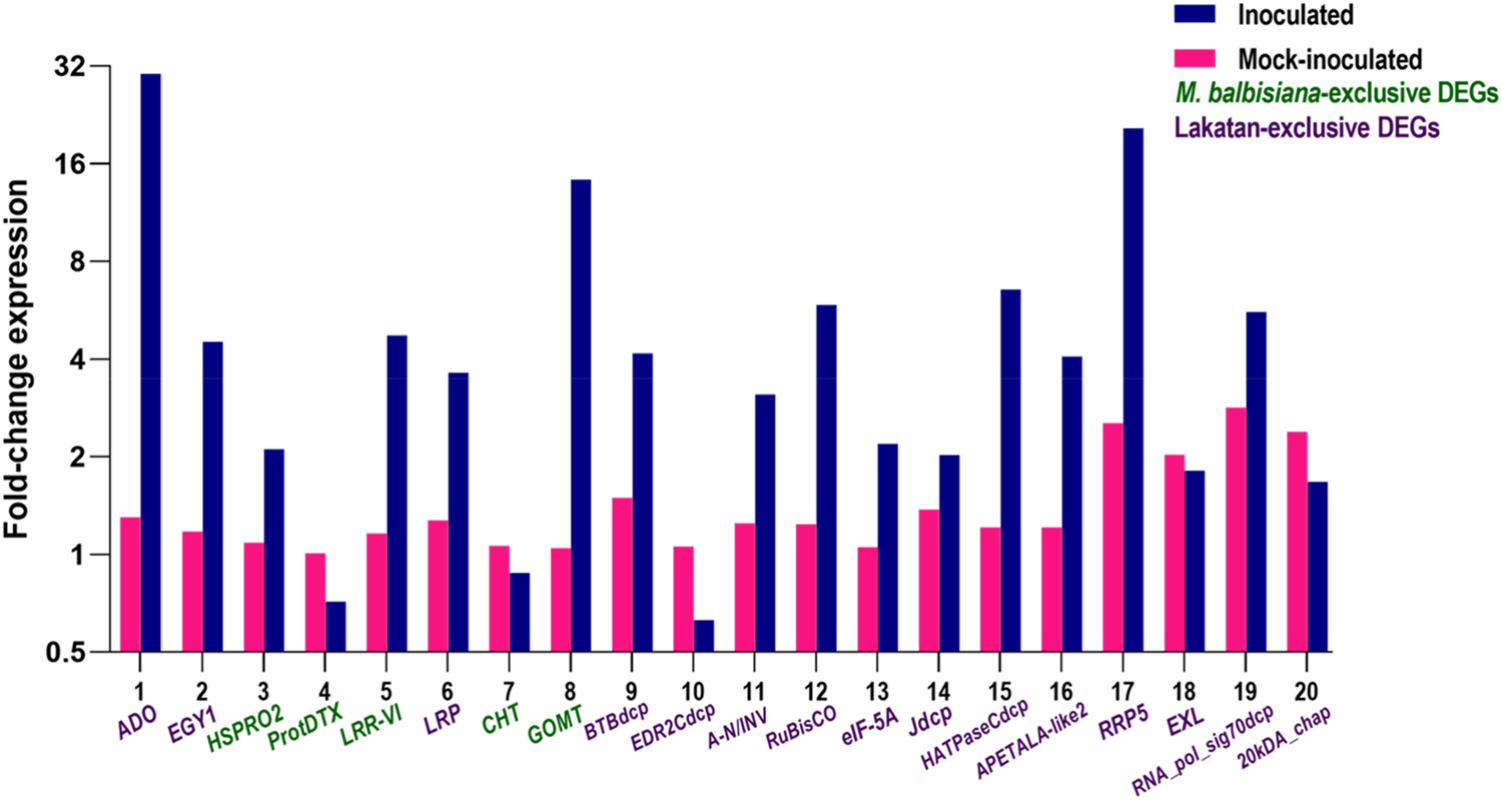
RT-qPCR expression profiles and validation of exclusive DEGs in wild *M. balbisiana* and ‘Lakatan’. *L2* gene was used as internal control^43^. *ADO:* Adagio-like protein (Ma07_g10990), *EGY1:* Probable zinc metalloprotease EGY1 (Ma06_g31610), *HSPRO2:* Nematode resistance protein-like HSPRO2 (Ma10_g05760), *ProtDTX:* protein detoxification (Ma07_g23140), *LRR-VI:* leucine rich repeat protein kinase VI (Ma07_g26720), *LRP:* Light regulated protein (Ma06_g30390), *CHT:* chitinase (Ma03_g28040), *GOMT:* Glucuronoxylan 4-O-methyltransferase (Ma08_g29650), *BTBdcp:* BTB domain-containing protein (Ma11_g13780), *EDR2dcp:* EDR2_C domain-containing protein (Ma02_g14310), *A-N/INV:* alkaline/neutral invertase (Ma08_g06150), *RuBisCo* (Ma11_g20430), *eIF-5A:* Eukaryotic translation initiation factor 5A-5 (Ma11_g09120), *Jdcp:* J domain-containing protein (Ma06_g32730), *HATPaseCdcp:* HATPase C domain-containing protein (Ma03_g29390), *APETALA-like2* (Ma03_g29391), *RRP5:* rRNA biogenesis protein RRP5 (Ma07_g07360), *EXL:* Protein EXORDIUM-like (Ma06_g32100), *RNA_pol_sig70dcp:* RNA_pol_sigma70 domain-containing protein (Ma11_g15590), *20kDA-chap:* 20kDA chaperonin, chloroplastic (Ma05_g04870).

RT-qPCR analysis was implemented to validate the findings of transcriptomic analysis. Sixteen DEGs were selected for primer design in RT-qPCR experiments to assess the accuracy of the comparative transcriptomic results (Supplementary Table S4). The RT-qPCR results demonstrated high concordance between the two methods for differential gene expression analysis, with 12 out of the 16 selected DEGs exhibiting consistent expression patterns. For instance, the gene expression of Adagio-like (*ADO*) protein, a candidate host factor gene for successful BBTV progression in banana, was found to be up-regulated in ‘Lakatan’. While RNA-Seq data showed an 5.3-fold change, RT-qPCR indicated a 30.31-fold change between mock-inoculated and BBTV-inoculated states at 72 hpi. The same pattern holds for a candidate resistance gene, the glucuronoxylan 4-O-methyltransferase gene, which may play a role in enhancing banana resistance through cell wall modification. Its expression values, as determined by RNA-seq analysis, exhibited a 3.72-fold increase, while RT-qPCR analysis also indicated an up-regulation, with a 14.28-fold change. To normalize gene expression, the *L2* gene transcript was used as an endogenous control^43^. Figure 2 illustrates the RT-qPCR data of the selected DEGs for validation.

## Discussion

### Insights into typical defense response of banana against BBTV

Regardless of the banana genotype, 62 genes are commonly differentially expressed and have similar patterns of gene expression upon the exposure of the host plant to the invading pathogen. The defense reaction of banana to BBTV, independent of whether a disease would arise or not, is represented by this shared gene set of both resistant and susceptible genotypes. To combat against invading pathogens, plants have many layers of defense^44^. Recognition of pathogen invasion is essential for triggering an effective plant defense response. To achieve this, plants possess pattern recognition receptors (PRRs) to recognize the evolutionary conserved pathogen's molecular fingerprints known as pathogen-associated molecular patterns (PAMPs) or microbe-associated molecular patterns (MAMPs) resulting in pattern-triggered immunity (PTI)^45–48^. All known plant PRRs are categorized as either receptor-like kinases (RLKs) or receptor-like proteins (RLPs) with functional domains, both of which are present in the plasma membrane^49^. In Arabidopsis, cysteine-rich receptor like kinase 6 (*CRK6*) overexpression increased the PTI response and developed resistance to *Pseudomonas syringae* pv. tomato DC3000 by associating with a PRR, the FLAGELLIN SENSING2 (*FLS2*)^50^. CaCRK6 was also observed to heterodimerized with CaCRK5, suggesting its role in the innate immune response of pepper against *Ralstonia solanacearum* infection^51^. Hence, the up-regulation of *CRK6* (Ma06_g23640) in both banana genotypes may indicate its potential function in the recognition of BBTV to initiate basal defense response.

Following the recognition of the pathogen via RLKs or RLPs, plants activate downstream signaling networks regulated by Ca^2+^-dependent protein kinase (CDPK) and mitogen-activated protein kinase (MAPK) cascades^52^. To trigger secondary and late defensive responses, MAPK activations may need to exceed a threshold in both duration and amplitude and interact differentially or synergistically with Ca^2+^ signaling^52, 53^. The activities and synthesis of various transcription factors (TFs), enzymes, hormones, peptides, and antimicrobial chemicals are regulated by MAPK and CDPK signaling networks in specific and overlapping manner that contribute to the defense response against various pathogens^52, 54–56^. Thus, the down-regulation of *MAPK* (Ma08_g21950) and the up-regulation of a *CDPK* (Ma06_g31170) gene expression in banana following the introduction of BBTV indicates a possible role in the regulation of processes essential to the defense response through kinase signaling pathways.

Light perception also contributes to the response of plants to pathogen attack by activating signaling pathways, regulating gene expression and controlling the cell death response^57, 58^. To sense light, plants utilize a group of proteins collectively known as photoreceptors^59^. In Arabidopsis, HYPERSENSITIVE RESPONSE TO TCV (HRT)-mediated plant defense against the *Turnip Crinkle Virus* (TCV) directly depends on blue-light photoreceptors, PHOT1 and PHOT2^58^. This process is negatively regulated by CONSTITUTIVE PHOTOMORPHOGENIC 1 (COP1), a primary regulator of light signaling, by interacting and tagging HRT protein for degradation via the 26S proteosome. The interaction between COP1 and SUPPRESSOR OF PHYA-105 (SPA) proteins determines the E3 ligase activity of COP1 in plants^60^. In this study, the two banana genotypes used were observed to have downregulation of the COP1-SPA adaptor subcomplex *COP1 *(Ma06_g36160) and two homologs of regulator component *SPA* (Ma01_g07220; Ma03_g32970). Hence, the downregulation of the components of potential key regulator, COP1-SPA E3 Ubiquitin Ligase complex, suggest the potential interaction among these genes to help in the typical defense of banana against BBTV.

The possible contribution of some HSPs to defend against the invading BBTV pathogen was also observed in both banana genotypes in which the expression of chaperone *HSP70* (Ma06_g36160) and co-chaperone *HSP40* (Ma04_g37990) were both observed to be downregulated*.* In other plant species, upregulation of *HSP70* was observed as a molecular mechanism in response to pathogens such as *Phytophthora parasitica* in tomato^61^ and *Blumeria graminis* in barley^62^. This is also consistent with the observed overexpression of *HSP40* in soybean and tomato, demonstrating its role in defense response^63, 64^.

### RNA-seq reveals putative susceptibility factors for BBTD progression in banana

Viruses heavily rely on the host’s machinery to promote the synthesis of vital components for viral replication and systemic cell-to-cell movement towards disease progression by hijacking various cellular processes^65, 66^. For instance, viruses tamper with the host translation system by preventing the recruitment of cap-dependent ribosomes for the host's mRNA translation (also known as "host shut-off"). Such viral strategy has an overall impact on the host’s anti-viral defense mechanism as well as promotes the synthesis of proteins for viral proliferation^29, 67^. A similar strategy was also implemented by the invading BBTV pathogen during its course of infection in ‘Lakatan’ banana cultivar based on the results of our current transcriptomic investigation. Components of the banana protein biosynthesis system such as 40S ribosomal protein SA, rRNA biogenesis protein/40S assembly factor RRP5, elongation factor 1α and 5α, and rRNA methyltransferase were all observed to be recruited by the invading pathogen to promote the biosynthesis of its own proteins for successful subversion of the host’s cells. Similar findings were obtained in a previous comparative transcriptomics study following *cucumber mosaic virus* (CMV) infection in the susceptible cucumber genotype, ‘Vanda’, where structural components of the ribosome and the translation biological process were substantially enriched and up-regulated^68^.

Viruses not only recruit plant translational components but also exploit and re-localize host genes involved in DNA replication and transcription to increase viral replication and spread^69, 70^. Consequently, genes in banana genome implicated in the RNA biosynthesis and processing are somewhat being mobilized by BBTV for its advantage, as evidenced by the ‘Lakatan’-specific overexpression of RNA polymerase sigma factor 70 and phosphorolytic exoribonuclease PNP. Most ssDNA viruses, such as BBTV, use the host’s DNA replication system to convert their ssDNA to intermediate dsDNA, which is subsequently transcribed into mRNA^71, 72^. Moreover, the RNA processing of the host cell, such as splicing machinery, is needed by the virus for the maturation of its mRNA necessary for proliferation, survival, and adaptation within the host^72^.

Virulent pathogens must overcome the initial layer of resistance of the host for successful infection, leading to disease development. In a bid to subdue PTI, these pathogens introduce effector proteins into the plant cell. With this mechanism, pathogens can survive and complete their life cycle, developing effector-triggered susceptibility (ETS) of the host^48, 73–75^. Plant virus effectors target various host cellular processes such as transcription factors regulating defense responses, protein degradation pathways, and re-localization of plant proteins^75^. Transcription factors classified as BBX-DBB, BBX-CO, DOF, MYB, REVEILLE, bZIP, NPL, ERF, AP2, NAC, and bHLH were revealed as differentially expressed in the susceptible cultivar during the BBTV infection process. Although no direct link has been established on how BBTV utilizes these host TFs via effectors, previous research shows that the association of betasatellites (βC1) with *tomato yellow leaf curl China virus* (TYLCCNV) increased viral pathogenicity by binding to several transcription factors that regulate plant defense responses^76^. Recently, it was reported that a self-replicating alphasatellite associated with BBTV contributes to viral replication, transcription, siRNA synthesis, and transmission^77^. Thus, more research should be undertaken to determine if the BBTV-associated alphasatellite interacts with the host's TFs to enhance viral pathogenicity in banana. Moreover, our results also suggest that the invading BBTV pathogen also recruits the host SCF (SKP1-CUL1-F-box protein) ubiquitin ligase complex, which typically functions for JA signaling and defense responses, to enhance the degradation and ubiquitination of its pathogenesis machinery via ubiquitin/26S proteasome system. Viruses achieve this mechanism via molecular mimicry in which the virus-encoded F-box protein interacts with the host SCF complex to attenuate plant antiviral defenses^36, 78^.

Heat shock proteins, particularly HSP70, are essential components of plant immunity, taking part in both PTI and ETI responses^79^. However, viruses use the HSPs of the host for their transcription, translation, post-translational modification and cellular localization^80^. When compared to the resistant banana genotype, six exclusively differentially expressed HSP genes was observed in the susceptible banana genotype while none on the resistant wild *M. balbisiana.* This is also further supported by the GO enrichment analysis in which the heat shock proteins involved in protein folding/refolding are significantly enriched in ‘Lakatan’ which is not observed in wild *M. balbisiana*. Hence, rather than aiding in plant immunity^21^, the HSPs in banana are being exploited by BBTV for its own benefit by recruiting them for enhanced viral invasion, replication, and maturation^21^. In response to viruses of resistant plants, the downregulation of HSP proteins was observed to restrict viral movement through plasmodesmata^81^. Therefore, silencing or inhibiting the expression of these HSP genes may contribute to preventing the progression of diseases caused by viral pathogens^82–84^.

### Differential transcriptomic analysis provided insights into resistance mechanisms of banana against BBTV

By comparing to the susceptible banana genotype, we were able to identify resistance genes that may contribute to the high degree of BBTV resistance reported in wild *M. balbisiana*^10^*.* Our work demonstrates that natural BBTV resistance found in the wild encompasses complex and multi-component mechanisms^85^. These include but are not limited to pathogen detection and response, action of phytohormones, reactive oxygen species (ROS), hypersensitive response (HR), and production of secondary metabolites.

Unlike the susceptible ‘Lakatan’ genotype, the resistant wild *M. balbisiana* was found to have a greater number of exclusively differentially expressed protein kinase genes [three in ‘Lakatan’ (Ma04_g37140, Ma07_g13740, Ma09_g06560) while six in wild *M. balbisiana* (Ma04_g09590, Ma04_g22180, Ma04_g25580, Ma07_g26720, Ma08_g26200, Ma11_g04550)], which may be involved in pathogen recognition and signaling, activating the overall resistance mechanisms in banana against BBTV. Protein kinases are essential components of signaling networks in plants that control numerous aspects of growth, development, and stress responses, including pathogen defense^86^. Protein kinases also play a role in other defense mechanisms, such as the production of reactive oxygen species (ROS) and the synthesis of defense-related hormones, such as salicylic acid (SA) and jasmonic acid (JA)^87^. Gene expression of wild *M. balbisiana*-exclusive protein kinase were all down-regulated except for Receptor-like protein kinase (Ma04_g22180). The downregulation of most protein kinases might trigger compensatory mechanisms in the resistant genotype. Other defense-related genes or pathways may be upregulated in response to the reduced activity of these set of protein kinases. Overall, the roles of protein kinases in plant immunity against viral pathogens are complex and multifaceted, involving various signaling pathways and defense mechanisms. However, further research is needed to fully comprehend the function of protein kinases in banana resistance to BBTV and to develop novel strategies for enhancing plant immunity by applying the findings of this current transcriptomic analysis to actual banana breeding applications, such as the development of EST-SSR markers tagging this candidate resistance gene analogs transcriptomic sequences^88^.

Restriction of further pathogen infection via HR and cell modification is apparently being implemented by the resistant banana genotype to protect itself against BBTV. HR mechanism in plants produces a containment zone via programmed cell death to prevent pathogens from spreading, which is an effective method for biotrophs such as viruses^85^. Hence, the over-expression of hypersensitive-induced response protein 1 gene (Ma07_g25320) in the resistant genotype provided insights on the possible role of this gene to resist further infection of BBTV. The contribution of hypersensitive-reaction protein in plant basal resistance against *Rice stripe virus*, *Turnip mosaic virus*, *Potato virus X*, and the bacterial pathogens *Pseudomonas syringae* and *Xanthomonas oryzae* via *EDS1* and salicylic acid-dependent pathways was previously demonstrated in *Nicotinia benthamiana* and rice^89^. Moreover, genes involved in cell wall organization were differentially expressed in the resistant cultivar based upon BBTV introduction, particularly those involved in xylan modification and degradation, cuticular lipid formation and biosynthesis of lignin. Various researches on essential plant defense cell wall-associated proteins in differential susceptible and resistance responses in plants demonstrate that cell wall organization (loosening and/or tightening) can influence viral propagation^90^. Hence, HR allows rapid, localized and programmed death of infected cells, while cell wall modification reinforces plant cell walls to prevent further viral invasion. These two mechanisms may act together as a defense strategy in wild *M. balbisiana* to combat BBTV invasion.

Secondary metabolites, on the other hand, have been implicated as elicitors of resistance against pests and pathogens in several plant species^91^. Interestingly, the resistant wild banana appears to be undergoing differential production of secondary metabolites in response to BBTV exposure, including terpenes, carotenes, and flavones as evidenced by the differential expression of genes for their biosynthesis. Plant secondary metabolites are involved in several biological processes, including innate immunity and defense signaling^92^. Volatile compounds such as terpenes and terpenoids are known to have deterrent effects to insect pests. However, the non-preference of banana aphids has been noted, but warrants further investigation on antixenosis resistance mechanisms. A study by Luan et al.^93^ relates the role of terpenoids in vector-virus mutualism, where *tomato yellow leaf curl China virus* (TYLCCNV) infection has suppressed terpenoid biosynthesis in tobacco, thus allowing its whitefly vectors to successfully infest a non-suitable host. Furthermore, studies have shown the role of terpenoid compounds through knock out assays. As an example, a terpenoid phytoalexin was reported to be involved in the basal response of *N. benthamina* against *Potato Virus X* (initially non-suitable host) catalyzed by terpenoid synthase 1 (TPS1)^94^, suggesting a potential role of the TPS DEGs in banana BBTV resistance*.* Additionally, cross-talk between flavones and salicylic acid reveals that flavone biosynthesis is involved in plant-pathogen interactions. Notably, wild banana downregulates the expression of *FNS* (Ma02_g12040), which is homologous to Arabidopsis DOWNY MILDEW RESISTANT6 (*AtDMR6*) and establishes interaction among flavones, hormone action and pathogen attack^95^. Therefore, these suggest the potential role of the aforementioned phytochemicals in basal resistance against BBTV in wild *M. balbisiana*. Nevertheless, further functional analysis and metabolic profiling will be required to validate these claims.

### Applications to banana breeding programs

Comparative transcriptomics is a powerful tool for identifying genes and pathways that are differentially expressed under different environmental conditions, including pathogen invasion. The application of this technique in the development of banana varieties that can resist BBTV infection can lead to a better understanding of the molecular mechanisms underlying the disease and the identification of genes and pathways that are involved in the host–pathogen interactions.

For the first time, this current research sheds insights into the fundamental understanding of the host-dependent infection process of the most destructive banana viral disease as well as unravels the natural resistance mechanism (such as pathogen recognition receptors, LRRs, and cell wall modifying enzymes) found in wild bananas. Comparative transcriptomic analysis was able to identify candidate resistance mechanisms in the wild *M. balbisiana* that might have been lost during the process of cultivation of commercial banana varieties for outstanding fruit quality traits. Thus, the identification and incorporation of the resistance alleles from the wild banana genotypes into the existing banana varieties is critical to developing DNA markers for use in marker-assisted plant breeding programs ^43^. Moreover, the potential host factors that are essential to the successful invasion of BBTV in banana that this research provides can be targeted for genome editing (e.g. CRISPR-Cas) towards creating new sources of resistance alleles towards development transgene-free banana varieties.

Moreover, the insights gained from comparative transcriptomics can be used to develop novel disease control strategies. For instance, by targeting genes and pathways that are involved in the pathogen invasion, researchers can develop new fungicides, biocontrol agents or other novel strategies that interrupt the BBTV life cycle.

## Materials and methods

### Preparation of biological samples and aphid-assisted inoculation

**Figure 3:**
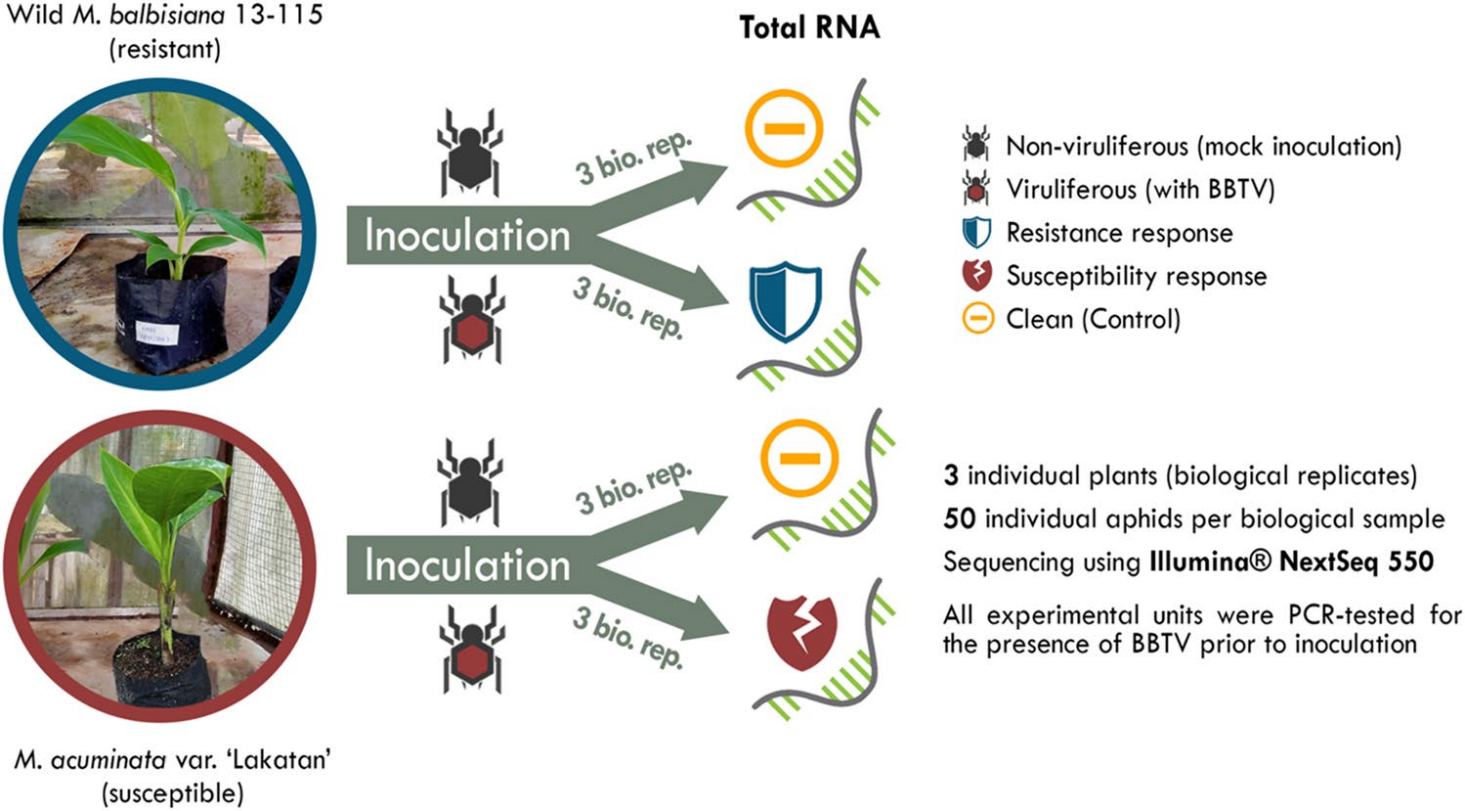
Schematic diagram depicting the biological materials and experimental treatments used for the generation of transcriptomic sequences.

BBTV-resistant wild *M. balbisiana*^9^ (Acc No. MB 13-155) conserved and maintained at the National Plant Genetic Resource Laboratory (NPGRL), Institute of Plant Breeding, University of the Philippines Los Baños (IPB-UPLB) and BBTV-susceptible *M. acuminata* ‘Lakatan’^6^ were used for the transcriptomic analysis (Fig. 3). The biological samples were grown in large pots filled with sterilized soil, and kept inside an insect-proof cage in a greenhouse at IPB-UPLB. The samples were tested using PCR detection^96^ to guarantee that the biological materials were free of BBTV infection. For the BBTV-challenged treatment, three biological replicates each of wild *M. balbisiana* and *M. acuminata* ‘Lakatan’ were inoculated with BBTV by transferring 50 individual viruliferous aphids (*P. nigronervosa*) to each plants. On the other hand, the mock-inoculated treatment was prepared by feeding 50 individual BBTV-negative non-viruliferous aphids to each of the three wild *M. balbisiana* seedlings and three *M. acuminata* ‘Lakatan’ plants. RNA samples were extracted from youngest leaf tissues of banana plants 72 h after inoculation (hpi).

### RNA extraction of BBTV-resistant and BBTV-susceptible banana accessions

Around 200 mg of plant tissues from each banana samples were homogenized in liquid nitrogen in double sterilized mortar and pestle. Total RNA from youngest leaf tissues of three biological replicates of wild *M. balbisiana* and *M. acuminata* ‘Lakatan’ (BBTV-inoculated and mock-inoculated) were extracted using the modified RNeasy Plant Mini Kit Protocol (QIAGEN, GmbH, Hilden, Germany).

RNA concentration and quality were determined by gel electrophoresis in 1% UltraPure™ agarose (Invitrogen Corp., Carlsbad, California, USA) in 1X TBE running buffer at 100 V for 30 min. The gel was stained with 0.1 µL Gel Red (Biotium, CA, USA) visualized under UV illumination at 300 nm using the Enduro GDS Touch Imaging System (Labnet International, Inc, Edison, New Jersey, USA). RNA was quantified using Qubit™ RNA HS Assay Kit (Life Technologies, Thermo Fisher Scientific Inc.) on Qubit™ 3.0 Fluorometer (Life Technologies, Thermo Fisher Scientific Inc.) following the manufacturer’s instruction.

### Next-generation sequencing of extracted RNA

High-quality RNA samples from wild *M. balbisiana* and *M. acuminata* ‘Lakatan’ were sent to the Philippine Genome Center—DNA Sequencing Core Facility (PGC-DSCF), University of the Philippines Diliman, Diliman, Quezon City, Philippines for next-generation sequencing. Quality control check was done through RNA quantitation, 260/280 and 260/230 absorbance measurements, and gel separation using Microchip Electrophoresis System (MCE™-202 MultiNA; Shimadzu Biotech, Kyoto, Japan). Sequencing libraries were constructed using TruSeq Stranded Total RNA Library Prep Plant (Illumina), followed by sequencing on the Illumina NextSeq 500/550 sequencer.

### Bioinformatics analysis

**Figure 4:**
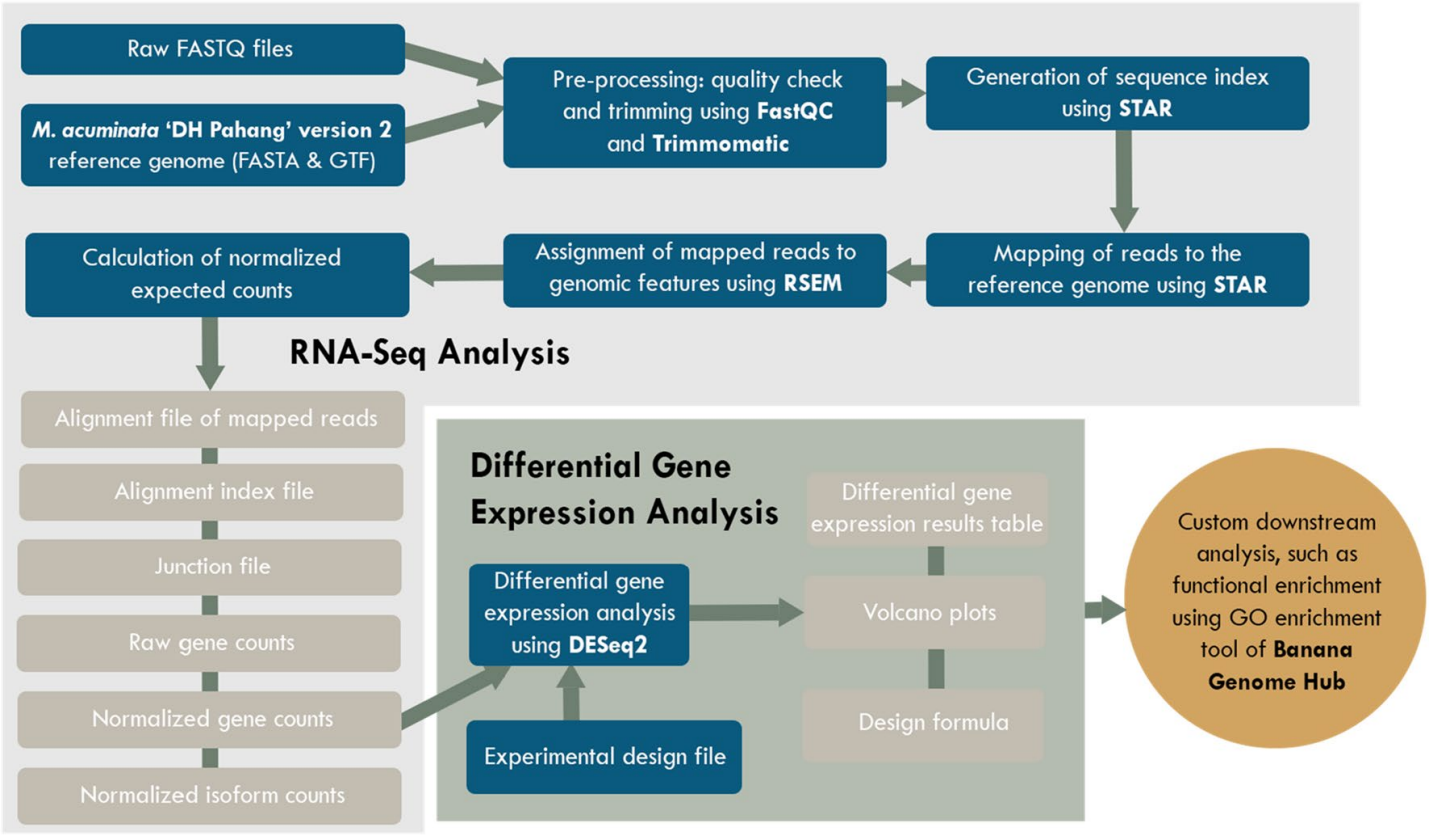
Overview of the bioinformatics processes implemented to compare the transcriptomic profiles of wild *M. balbisiana* and ‘Lakatan’ in response to BBTV-inoculation using mock-inoculated samples as baseline control.

The paired-end raw transcriptomic sequences were pre-processed by removing the adapter sequences and low-quality base score nucleotide sequences using *Trimmomatic* v0.36^97^ with the following parameters: SLIDINGWINDOW:4:15 MINLEN:50. The paired-end reads were mapped to the *M. acuminata* version 2 genome^12^ using *STAR* v2.7.10a^98^ with the following settings: --outFilterMatchNmin 0 --outFilterScoreMinOverLread 0.3 --outFilterMatchNminOverLread 0.3 --quantMode TranscriptomeSAM. The resulting binary alignment map files were quantified using rsem-calculate-expression function of *RSEM*^99^ using the following parameters: -p 60 --paired-end. A matrix of the read count data generated from RSEM quantification was prepared and imported to R package *DESeq2* v1.26.0^13^ using *tximport* v1.20.0^100^. The overview of the bioinformatics processes implemented in the analysis is presented in Fig. 4.

A single-factor design was implemented independently for each genotype (wild *M. balbisiana* and ‘Lakatan) to consider the factor introduced by the differences in the inoculation state (BBTV-inoculated vs. mock-inoculated) from biological replicates. After normalization using the relative log normalization (RLE) method, the DEGs between the two treatments were identified. The Wald test from the general linear model (GLM) fitting was used to determine* p*-values. False discovery rate (FDR) values were derived using the Benjamini–Hochberg *p*-value adjustment algorithm. Genes with adjusted *p*-value (*padj*) < 0.05 were considered differentially expressed between BBTV-inoculated and mock-inoculated ‘Lakatan’ and wild *M. balbisiana*. Gene ontology enrichment analysis was performed using the GO enrichment tool from Banana Genome Hub (https://banana-genome-hub.southgreen.fr/content/go-enrichment), with pvalue and qvalue cutoffs of 0.05 and 0.1, respectively.

### Validation of differentially expressed genes (DEGs) via RT-qPCR

Leaf samples from three biological replicates of mock-inoculated and BBTV-inoculated (72 hpi) wild *M. balbisiana* and ‘Lakatan' were collected and isolated using the Monarch® Total RNA Miniprep Kit (New England Biolabs, Inc.) manufacturer’s protocol for tissues. The isolated RNA samples were then subjected to cDNA synthesis using the SuperScript™ III First-Strand Synthesis System (Thermo Scientific, Inc.), following the manufacturer’s protocols. Synthesized cDNA were stored to − 20 °C and were used as templates for RT-qPCR validation experiments.

Expression of the DEGs from the RNA-Seq data were validated using RT-qPCR amplification. qPCR primers for the target genes are presented in Supplementary Table S4. Detection steps were performed using Bio-Rad CFX96 Real-Time PCR Detection System (Bio-Rad Laboratories, Inc., Hercules, California, USA). Three technical replicates for each of the biological groups of wild *M. balbisiana* and ‘Lakatan’ were prepared in accordance with the MIQE guidelines for qPCR^101^. Reactions of 10 μL total volume consisted of 5 µL 1 × Universal SYBR® Green Supermix (Bio-Rad Laboratories, Inc.), 0.20 µL 0.20 µM of each DEG primer pair, and 1 µL of cDNA. *L2* was used as the internal control gene for DEGs validation of the primers^43^. The qPCR parameters used for the primers were as follows: 95 °C for 3 min, 40 cycles of 95 °C for 15 s, optimized primer annealing temperature for 30 s and a default instrument protocol for the melt curve. To determine the relative gene expression of the identified DEGs, quantification cycle values obtained from the RT-qPCR runs were computed using the Livak method (2^−ΔΔCq^).

### Research involving plants

The authors declare that all local, national or international guidelines and legislation were adhered for the use of plants in this study. The wild *M. balbisiana*^9^ (MB 13-155/GB61996) plants used in this research were requested from NPGRL, IPB-UPLB, a national public repository of important and potentially useful agricultural crops including the wild and weedy relatives. It was previously collected and identified by Dr. Lavernee S. Gueco and Michael B. Biguelme of NPGRL, IPB-UPLB. A voucher specimen (CAHUP 74278) was deposited to a public herbarium at the Museum of National History (MNH), University of the Philippines Los Baños, College, Laguna, Philippines. Meanwhile, *Musa acuminata *‘Lakatan' is a commercial banana variety that is highly popular in the local markets of the Philippines.

### Supplementary Information

Supplementary Table S1.

Supplementary Table S2.

Supplementary Table S3.

Supplementary Table S4.

## Data Availability

The RNA-seq datasets generated in this study have been submitted to National Center for Biotechnology Information (NCBI) under Accession No. PRJNA746416.
