# Supplementary material for: Comparative RNA-seq analysis of resistant and susceptible banana genotypes reveals molecular mechanisms in response to banana bunchy top virus (BBTV) infection
Source: Sci Rep. 2023 Oct 31;13:18719. doi: 10.1038/s41598-023-45937-z (PMC10618458; doi:10.1038/s41598-023-45937-z)
Supplement: Supplementary file 1 — Supplementary Table S1. [file 41598_2023_45937_MOESM1_ESM.pdf]

Comparative RNA-seq analysis of resistant and susceptible banana genotypes reveals molecular mechanisms in response to *Banana bunchy top virus* (BBTV) infection

Darlon V. Lantican, Jen Daine L. Nocum, Anand Noel C. Manohar, Jay-Vee S. Mendoza, Roanne R. Gardoce, Grace C. Lachica, Lavernee S. Gueco and Fe M. Dela Cueva

Supplementary Table S1. Statistics for raw and trimmed transcriptome reads, as well as reference-based mapping statistics, from wild *Musa balbisiana* and 'Lakatan' mock- and BBTV-inoculated samples to the Musa acuminata DH Pahang v2 reference genome.

NGS reads / Mapping Statistics	CLAK1	CLAK2	CLAK3	XLAK1	XLAK2	XLAK3	CMB1	CMB2	CMB3	CMB3	XMB1	XMB2	XMB3
Number of spots	17632235	27722302	30665733	20430184	24407252	17038836	24333650	11787694	20716804	20716804	22794698	15479847	16448751
Number of paired-end reads per sample	35264470	55444604	61331466	40860368	48814504	34077672	48667300	23575388	41433608	41433608	45589396	30959694	32897502
Average input read length	148	148	150	148	148	147	148	147	148	148	148	147	148
Number of mapped reads	16590717	26148659	29214097	19213971	22997174	16100944	22864616	11138840	19578621	19578621	21694114	14796256	15575661
Mapped reads %	94.09	94.32	95.27	94.05	94.22	94.50	93.96	94.50	94.51	94.51	95.17	95.58	94.69
Number of uniquely mapped reads	8690772	14004833	13833031	8009272	12925319	8229585	12498723	4974044	10114860	10114860	9485266	7083483	7135850
Uniquely mapped reads %	49.29	50.52	45.11	39.2	52.96	48.3	51.36	42.2	48.82	48.82	41.61	45.76	43.38
Average mapped length	124.2	122.93	126.56	125.33	120.46	122.46	122.78	120.7	121.73	121.73	123.78	120.54	123.86
Mismatch rate per base, %	3.8	3.84	3.22	4	4	3.87	3.83	4.46	4.04	4.04	4.17	4.29	4.26
Deletion rate per base	0.06	0.09	0.06	0.06	0.1	0.07	0.09	0.07	0.09	0.09	0.07	0.09	0.07
Deletion average length	2.47	2.64	2.26	2.29	2.68	2.48	2.56	2.46	2.63	2.63	2.31	2.55	2.38
Insertion rate per base	0.04	0.05	0.05	0.06	0.05	0.04	0.05	0.06	0.06	0.06	0.07	0.05	0.07
Insertion average length	2.59	2.66	2.94	2.68	2.69	2.42	2.68	2.26	2.75	2.75	2.67	2.54	2.67
Number of reads mapped to multiple loci	7899945	12143826	15381066	11204699	10071855	7871359	10365893	6164796	9463761	9463761	12208848	7712773	8439811
% of reads mapped to multiple loci	44.8	43.81	50.16	54.84	41.27	46.2	42.6	52.3	45.68	45.68	53.56	49.82	51.31
% of reads unmapped: too short	5.75	6.84	4.63	5.78	5.63	5.33	5.9	5.26	5.33	5.33	4.66	4.24	5.13
