# Supplementary material for: Comparative RNA-seq analysis of resistant and susceptible banana genotypes reveals molecular mechanisms in response to banana bunchy top virus (BBTV) infection
Source: Sci Rep. 2023 Oct 31;13:18719. doi: 10.1038/s41598-023-45937-z (PMC10618458; doi:10.1038/s41598-023-45937-z)
Supplement: Supplementary file 2 — Supplementary Table S2. [file 41598_2023_45937_MOESM2_ESM.pdf]

Comparative RNA-seq analysis of resistant and susceptible banana genotypes reveals molecular mechanisms in response to *Banana bunchy top virus* (BBTV)

Darlon V. Lantican, Jen Daine L. Nocum, Anand Noel C. Manohar, Jay-Vee S. Mendoza, Roanne R. Gardoce, Grace C. Lachica, Lavernee S. Gueco and Fe M. Dela Cueva

Supplementary Table S2. List of differentially-expressed genes between mock- and BBTV-inoculated BBTV-resistant wild *M. balbisiana* and BBTV-susceptible Lakatan.

Genotype	Gene ID	baseMean	log2FoldChange	lfcSE	stat	pvalue	padj
wild *M. balbisiana*	Ma01_g02150	64.1770764	-4.1310949	0.5587927	-7.3928934	1.44E-13	1.24E-09
wild *M. balbisiana*	Ma03_g29410	111.082278	-4.5646531	0.66362582	-6.8783537	6.05E-12	2.61E-08
wild *M. balbisiana*	Ma06_g21680	199.352333	-2.6640556	0.39644503	-6.7198613	1.82E-11	5.22E-08
wild *M. balbisiana*	Ma11_g07100	89.6108721	-2.6708497	0.41614776	-6.4180321	1.38E-10	2.97E-07
wild *M. balbisiana*	Ma10_g20080	56.2446043	-4.9641309	0.78239901	-6.3447562	2.23E-10	3.83E-07
wild *M. balbisiana*	Ma07_g20820	144.536172	-3.2338113	0.51765189	-6.2470773	4.18E-10	5.60E-07
wild *M. balbisiana*	Ma09_g27880	42.3813689	3.4378111	0.55149569	6.23361373	4.56E-10	5.60E-07
wild *M. balbisiana*	Ma06_g12350	59.8285923	3.43367038	0.55987849	6.132885	8.63E-10	9.28E-07
wild *M. balbisiana*	Ma02_g08820	819.29753	-2.2545341	0.3897269	-5.7849075	7.26E-09	6.37E-06
wild *M. balbisiana*	Ma10_g15170	76.9777422	-3.5857892	0.62021027	-5.7815702	7.40E-09	6.37E-06
wild *M. balbisiana*	Ma11_g14200	30.1277579	-4.1338625	0.73210412	-5.64655	1.64E-08	1.20E-05
wild *M. balbisiana*	Ma01_g01670	98.5147458	-2.9739107	0.52828535	-5.6293642	1.81E-08	1.20E-05
wild *M. balbisiana*	Ma06_g33250	39.944845	-5.4750213	0.97267373	-5.6288364	1.81E-08	1.20E-05
wild *M. balbisiana*	Ma05_g15310	39.4872802	-3.5378061	0.63092088	-5.6073688	2.05E-08	1.26E-05
wild *M. balbisiana*	Ma09_g29480	22.0327093	-21.857851	3.90885747	-5.5918771	2.25E-08	1.29E-05
wild *M. balbisiana*	Ma05_g21440	17.3282239	-21.510792	3.90942612	-5.5022889	3.75E-08	2.02E-05
wild *M. balbisiana*	Ma10_g31030	78.2677871	-2.541962	0.46417443	-5.4763078	4.34E-08	2.20E-05
wild *M. balbisiana*	Ma04_g31330	176.30014	-2.3641732	0.43937834	-5.3807231	7.42E-08	3.55E-05
wild *M. balbisiana*	Ma01_g22550	70.9982339	-3.2460957	0.60793163	-5.3395736	9.32E-08	4.02E-05
wild *M. balbisiana*	Ma10_g14160	49.892229	-4.0492449	0.75841676	-5.3390763	9.34E-08	4.02E-05
wild *M. balbisiana*	Ma10_g28290	31.2427358	-4.0812311	0.77901766	-5.2389456	1.61E-07	6.62E-05
wild *M. balbisiana*	Ma04_g25580	76.5153875	-3.2721869	0.62768324	-5.2131182	1.86E-07	7.26E-05
wild *M. balbisiana*	Ma01_g07220	64.3146045	-3.1999113	0.6178825	-5.1788347	2.23E-07	8.23E-05

Comparative RNA-seq analysis of resistant and susceptible banana genotypes reveals molecular mechanisms in response to *Banana bunchy top virus* (BBTV)

Darlon V. Lantican, Jen Daine L. Nocum, Anand Noel C. Manohar, Jay-Vee S. Mendoza, Roanne R. Gardoce, Grace C. Lachica, Lavernee S. Gueco and Fe M. Dela Cueva

Supplementary Table S2. List of differentially-expressed genes between mock- and BBTV-inoculated BBTV-resistant wild *M. balbisiana* and BBTV-susceptible Lakatan.

wild *M. balbisiana*	Ma02_g21520	26.5109289	-4.2972326	0.83059177	-5.1736999	2.30E-07	8.23E-05
wild *M. balbisiana*	Ma07_g18310	827.689425	-3.2509965	0.63034832	-5.1574604	2.50E-07	8.62E-05
wild *M. balbisiana*	Ma08_g10850	36.1365998	-4.1093409	0.79998499	-5.1367725	2.79E-07	9.25E-05
wild *M. balbisiana*	Ma09_g08490	53.8189693	-3.9256685	0.77438269	-5.0694166	3.99E-07	0.0001272
wild *M. balbisiana*	Ma08_g23810	53.8020724	-2.7258564	0.53869215	-5.0601376	4.19E-07	0.00012878
wild *M. balbisiana*	Ma08_g10940	70.1766516	-4.1101058	0.8170393	-5.0304873	4.89E-07	0.0001452
wild *M. balbisiana*	Ma04_g33860	33.8542468	-3.1191132	0.62117068	-5.0213464	5.13E-07	0.00014721
wild *M. balbisiana*	Ma04_g16680	73.5285824	-2.8421081	0.56802269	-5.0035115	5.63E-07	0.0001563
wild *M. balbisiana*	Ma11_g00800	141.281267	-2.0046353	0.40700369	-4.9253493	8.42E-07	0.00022646
wild *M. balbisiana*	Ma05_g03580	49.1784943	-3.7388147	0.76002009	-4.919363	8.68E-07	0.00022646
wild *M. balbisiana*	Ma05_g27420	118.27708	2.57582667	0.52428384	4.91303843	8.97E-07	0.00022701
wild *M. balbisiana*	Ma07_g21230	109.742042	-2.0474737	0.4188265	-4.8885964	1.02E-06	0.00024974
wild *M. balbisiana*	Ma07_g26010	56.1453861	-2.2425096	0.46536984	-4.8187687	1.44E-06	0.00034535
wild *M. balbisiana*	Ma02_g24860	39.879426	-2.7545801	0.58251409	-4.7287785	2.26E-06	0.00052543
wild *M. balbisiana*	Ma02_g23730	49.2495896	2.46017154	0.5308237	4.63463014	3.58E-06	0.00080991
wild *M. balbisiana*	Ma10_g05590	63.8004874	-3.2769503	0.70806165	-4.6280579	3.69E-06	0.0008146
wild *M. balbisiana*	Ma08_g25160	230.870646	-1.7484528	0.37884808	-4.6151818	3.93E-06	0.0008451
wild *M. balbisiana*	Ma08_g07100	42.1019694	2.6618468	0.58241341	4.57037346	4.87E-06	0.00099644
wild *M. balbisiana*	Ma06_g36160	33.7488101	-2.4804897	0.54327808	-4.5657828	4.98E-06	0.00099644
wild *M. balbisiana*	Ma05_g06140	30.5364535	-5.8113408	1.27282417	-4.5657059	4.98E-06	0.00099644
wild *M. balbisiana*	Ma04_g00790	105.578302	-1.7886367	0.39250021	-4.5570338	5.19E-06	0.00101486
wild *M. balbisiana*	Ma06_g35530	102.477384	2.86424716	0.62973199	4.54835901	5.41E-06	0.0010341
wild *M. balbisiana*	Ma03_g11730	210.402189	-3.0897778	0.68072503	-4.5389514	5.65E-06	0.00105781
wild *M. balbisiana*	Ma11_g01360	16.7793319	-5.5002992	1.21871728	-4.5131871	6.39E-06	0.00116679

Supplementary Table S2. List of differentially-expressed genes between mock- and BBTV-inoculated BBTV-resistant wild *M. balbisiana* and BBTV-susceptible Lakatan.

wild *M. balbisiana*	Ma04_g18850	33.2707132	-2.4548463	0.54472337	-4.5065925	6.59E-06	0.00116679
wild *M. balbisiana*	Ma04_g21660	52.1684476	-2.8862009	0.64069016	-4.504831	6.64E-06	0.00116679
wild *M. balbisiana*	Ma06_g23640	89.9505546	2.19284619	0.49049908	4.47064287	7.80E-06	0.00134243
wild *M. balbisiana*	Ma04_g21890	55.3285096	-2.5858258	0.58209357	-4.4422855	8.90E-06	0.00150215
wild *M. balbisiana*	Ma09_g14900	287.820123	-3.2286578	0.7281954	-4.4337795	9.26E-06	0.00153263
wild *M. balbisiana*	Ma07_g23140	38.7611579	-2.9731604	0.67186295	-4.4252484	9.63E-06	0.00155048
wild *M. balbisiana*	Ma03_g20940	44.8992784	-2.2643135	0.51192447	-4.4231398	9.73E-06	0.00155048
wild *M. balbisiana*	Ma11_g20650	451.150817	-1.6752872	0.38165082	-4.389581	1.14E-05	0.00173473
wild *M. balbisiana*	Ma03_g15660	262.150251	2.89001848	0.65851451	4.38869372	1.14E-05	0.00173473
wild *M. balbisiana*	Ma02_g08850	95.6792166	-2.8612435	0.65219781	-4.3870793	1.15E-05	0.00173473
wild *M. balbisiana*	Ma11_g10900	39.0375292	-3.0182428	0.69314419	-4.3544227	1.33E-05	0.00197988
wild *M. balbisiana*	Ma01_g21040	8.49928277	-6.3564906	1.46839777	-4.3288615	1.50E-05	0.0021865
wild *M. balbisiana*	Ma01_g09220	36.291323	2.08749301	0.48420727	4.31115585	1.62E-05	0.00232968
wild *M. balbisiana*	Ma08_g26200	113.174847	-2.1389973	0.49876528	-4.288585	1.80E-05	0.00251333
wild *M. balbisiana*	Ma07_g25830	18.9714656	-3.239309	0.75559998	-4.2870686	1.81E-05	0.00251333
wild *M. balbisiana*	Ma03_g13370	52.2395279	-2.1745563	0.5103544	-4.2608751	2.04E-05	0.00272504
wild *M. balbisiana*	Ma09_g09640	25.8886868	-2.8446852	0.66784515	-4.2594981	2.05E-05	0.00272504
wild *M. balbisiana*	Ma05_g01120	178.805756	-2.717496	0.63813321	-4.2585091	2.06E-05	0.00272504
wild *M. balbisiana*	Ma05_g15800	649.652084	-2.4992907	0.58869126	-4.2455034	2.18E-05	0.00284426
wild *M. balbisiana*	Ma05_g07800	120.058889	-2.2212467	0.52446068	-4.2352969	2.28E-05	0.00293216
wild *M. balbisiana*	Ma06_g31170	24.9154329	3.12343389	0.73961648	4.22304526	2.41E-05	0.00305072
wild *M. balbisiana*	Ma09_g25280	40.3510289	2.88567083	0.6862118	4.20521887	2.61E-05	0.00325357
wild *M. balbisiana*	Ma09_g02300	349.571573	-1.6350932	0.38975917	-4.1951373	2.73E-05	0.00335312
wild *M. balbisiana*	Ma09_g07420	74.1921375	-1.9248774	0.46134017	-4.1723603	3.01E-05	0.00361111

Comparative RNA-seq analysis of resistant and susceptible banana genotypes reveals molecular mechanisms in response to *Banana bunchy top virus* (BBTV)

Darlon V. Lantican, Jen Daine L. Nocum, Anand Noel C. Manohar, Jay-Vee S. Mendoza, Roanne R. Gardoce, Grace C. Lachica, Lavernee S. Gueco and Fe M. Dela Cueva

Supplementary Table S2. List of differentially-expressed genes between mock- and BBTV-inoculated BBTV-resistant wild *M. balbisiana* and BBTV-susceptible Lakatan.

wild *M. balbisiana*	Ma06_g38570	45.6701345	-2.2791077	0.54630065	-4.1718927	3.02E-05	0.00361111
wild *M. balbisiana*	Ma10_g25380	414.452171	-1.6119878	0.39005684	-4.1326997	3.59E-05	0.00422718
wild *M. balbisiana*	Ma04_g16180	13.3388711	3.4895174	0.84540702	4.12761818	3.67E-05	0.00425085
wild *M. balbisiana*	Ma09_g30860	272.529787	1.82923115	0.44342842	4.12520048	3.70E-05	0.00425085
wild *M. balbisiana*	Ma04_g10370	20.7088766	-2.7180339	0.66032643	-4.1161973	3.85E-05	0.00432601
wild *M. balbisiana*	Ma11_g19880	29.9356289	-2.9723657	0.72230723	-4.1150988	3.87E-05	0.00432601
wild *M. balbisiana*	Ma01_g04010	30.6425912	-2.4797969	0.60489583	-4.0995437	4.14E-05	0.00456795
wild *M. balbisiana*	Ma06_g30910	70.4984291	-2.6789233	0.65454709	-4.0927893	4.26E-05	0.00464359
wild *M. balbisiana*	Ma04_g34180	613.53365	-1.7455322	0.42744339	-4.0836571	4.43E-05	0.00476962
wild *M. balbisiana*	Ma00_g00510	15.6969935	3.41368818	0.83672719	4.07981026	4.51E-05	0.00478937
wild *M. balbisiana*	Ma08_g23180	502.13989	3.06225702	0.75269211	4.06840588	4.73E-05	0.00496853
wild *M. balbisiana*	Ma11_g09710	106.63203	-6.0259106	1.48771907	-4.0504358	5.11E-05	0.00530132
wild *M. balbisiana*	Ma08_g21950	10.0441418	-5.6160447	1.38862747	-4.0443134	5.25E-05	0.00533334
wild *M. balbisiana*	Ma07_g08090	45.9313103	-1.9984261	0.49423784	-4.0434502	5.27E-05	0.00533334
wild *M. balbisiana*	Ma02_g19620	39.5884555	2.30553694	0.571046	4.03739266	5.40E-05	0.00540926
wild *M. balbisiana*	Ma10_g05760	34.8990676	-2.8543659	0.7084728	-4.0288997	5.60E-05	0.00554395
wild *M. balbisiana*	Ma09_g03430	31.6699673	2.32069302	0.57657743	4.024946	5.70E-05	0.00554998
wild *M. balbisiana*	Ma04_g39130	43.461532	3.25860658	0.80993441	4.02329687	5.74E-05	0.00554998
wild *M. balbisiana*	Ma03_g03480	76.4497645	3.18557853	0.79274477	4.01841632	5.86E-05	0.00560322
wild *M. balbisiana*	Ma09_g30010	41.1111031	2.36487112	0.59052893	4.00466601	6.21E-05	0.00587406
wild *M. balbisiana*	Ma08_g09190	16.2900629	2.90482326	0.72629271	3.99952141	6.35E-05	0.00593796
wild *M. balbisiana*	Ma03_g14330	12.2008166	-4.0732528	1.02043512	-3.9916823	6.56E-05	0.00602534
wild *M. balbisiana*	Ma01_g01110	7.78963856	-6.2064346	1.55512105	-3.9909656	6.58E-05	0.00602534
wild *M. balbisiana*	Ma10_g11570	20.6425875	-3.884626	0.97407875	-3.9879999	6.66E-05	0.00603692

Comparative RNA-seq analysis of resistant and susceptible banana genotypes reveals molecular mechanisms in response to *Banana bunchy top virus* (BBTV)

Darlon V. Lantican, Jen Daine L. Nocum, Anand Noel C. Manohar, Jay-Vee S. Mendoza, Roanne R. Gardoce, Grace C. Lachica, Lavernee S. Gueco and Fe M. Dela Cueva

Supplementary Table S2. List of differentially-expressed genes between mock- and BBTV-inoculated BBTV-resistant wild *M. balbisiana* and BBTV-susceptible Lakatan.

wild *M. balbisiana*	Ma01_g14940	198.655959	-2.0588959	0.51682523	-3.9837373	6.78E-05	0.00608227
wild *M. balbisiana*	Ma03_g32970	38.3963567	-2.6864491	0.67825411	-3.9608298	7.47E-05	0.00662737
wild *M. balbisiana*	Ma04_g27510	56.8695415	-3.2498724	0.8215975	-3.955553	7.64E-05	0.00670624
wild *M. balbisiana*	Ma09_g14720	91.4159316	-2.23122	0.56505999	-3.9486428	7.86E-05	0.00683305
wild *M. balbisiana*	Ma06_g38530	11.3254158	-4.309366	1.09268791	-3.9438215	8.02E-05	0.00690224
wild *M. balbisiana*	Ma07_g09610	213.092432	2.38507813	0.6057004	3.93771926	8.23E-05	0.00701
wild *M. balbisiana*	Ma08_g17730	14.0168528	-5.2311994	1.33764327	-3.9107582	9.20E-05	0.00772278
wild *M. balbisiana*	Ma09_g17760	108.05288	-1.5804105	0.40423015	-3.9096799	9.24E-05	0.00772278
wild *M. balbisiana*	Ma03_g20340	78.4069792	-2.1264058	0.54977103	-3.8678026	0.00010982	0.0090887
wild *M. balbisiana*	Ma03_g02450	39.6416239	-2.0551895	0.5317608	-3.864876	0.00011115	0.00911077
wild *M. balbisiana*	Ma02_g01300	12.43329	-5.0349772	1.31433937	-3.8308046	0.00012772	0.01036619
wild *M. balbisiana*	Ma11_g03670	14.4984214	3.89502548	1.01734752	3.82860861	0.00012887	0.01036619
wild *M. balbisiana*	Ma09_g08110	62.7072916	1.99347169	0.52164975	3.82147544	0.00013266	0.01057192
wild *M. balbisiana*	Ma09_g00790	24.7321132	-2.7919198	0.73443581	-3.8014484	0.00014385	0.01135908
wild *M. balbisiana*	Ma04_g22260	19.8043985	-2.4917819	0.65598868	-3.7985135	0.00014557	0.01138992
wild *M. balbisiana*	Ma09_g01360	23.0771167	-2.3233784	0.61205178	-3.7960488	0.00014702	0.01140005
wild *M. balbisiana*	Ma06_g16330	10.4781021	-4.2525948	1.12333096	-3.7857007	0.00015328	0.01177899
wild *M. balbisiana*	Ma09_g25900	44.8997058	-2.4951884	0.65982068	-3.781616	0.00015581	0.01186803
wild *M. balbisiana*	Ma03_g03280	36.793316	-3.1514676	0.83588876	-3.7701997	0.00016312	0.01231533
wild *M. balbisiana*	Ma11_g04550	68.0809333	-1.7409975	0.46357934	-3.7555546	0.00017296	0.01294478
wild *M. balbisiana*	Ma08_g22450	28.0770203	2.02257164	0.54113637	3.73763757	0.00018576	0.01378288
wild *M. balbisiana*	Ma04_g27610	625.459575	-1.1916353	0.32076525	-3.7149763	0.00020322	0.0149499
wild *M. balbisiana*	Ma05_g28720	10.1231552	-3.779012	1.02275909	-3.694919	0.00021996	0.01593059
wild *M. balbisiana*	Ma11_g02330	27.6486461	-2.3361004	0.63230573	-3.6945741	0.00022026	0.01593059

Supplementary Table S2. List of differentially-expressed genes between mock- and BBTV-inoculated BBTV-resistant wild *M. balbisiana* and BBTV-susceptible Lakatan.

wild *M. balbisiana*	Ma02_g05590	354.799881	1.46643183	0.39759485	3.68825664	0.0002258	0.01619521
wild *M. balbisiana*	Ma04_g20350	39.292898	-2.2525415	0.61170239	-3.6824141	0.00023104	0.01643409
wild *M. balbisiana*	Ma01_g21420	70.4225525	-2.2219739	0.60751259	-3.6574945	0.00025469	0.01796836
wild *M. balbisiana*	Ma07_g26720	33.5454087	-2.4030163	0.65900318	-3.6464412	0.0002659	0.01855364
wild *M. balbisiana*	Ma06_g36640	19.4591539	2.63335037	0.72243804	3.64508821	0.0002673	0.01855364
wild *M. balbisiana*	Ma06_g29050	213.465882	-2.5036305	0.68916741	-3.6328335	0.00028033	0.01930212
wild *M. balbisiana*	Ma01_g11670	92.3250577	2.38008504	0.65634585	3.62626662	0.00028755	0.0196423
wild *M. balbisiana*	Ma07_g27820	203.742547	-1.7257904	0.4763653	-3.6228298	0.0002914	0.0197485
wild *M. balbisiana*	Ma11_g02690	9.53965742	-4.5480635	1.25817429	-3.614812	0.00030057	0.02013255
wild *M. balbisiana*	Ma03_g03930	2076.02076	-1.3098166	0.36244862	-3.6137994	0.00030174	0.02013255
wild *M. balbisiana*	Ma10_g27530	9.65828424	4.40364073	1.22270368	3.60156005	0.00031631	0.02094238
wild *M. balbisiana*	Ma10_g12100	20.0149746	-4.2443968	1.18212066	-3.5904937	0.00033005	0.02168518
wild *M. balbisiana*	Ma06_g26470	41.6237231	-2.4632525	0.68671176	-3.5870253	0.00033447	0.02180909
wild *M. balbisiana*	Ma06_g35790	27.3185106	2.36465376	0.6612774	3.57588776	0.00034904	0.02258796
wild *M. balbisiana*	Ma03_g32890	74.6091634	-1.5764659	0.44130515	-3.5722807	0.00035389	0.02273056
wild *M. balbisiana*	Ma07_g25320	99.473165	1.46756688	0.41139338	3.56730798	0.00036067	0.02299456
wild *M. balbisiana*	Ma11_g05710	86.1157713	1.64065227	0.4604194	3.56338649	0.0003661	0.02316824
wild *M. balbisiana*	Ma04_g25840	318.048492	-1.8442958	0.51808909	-3.5598044	0.00037113	0.02316824
wild *M. balbisiana*	Ma10_g21920	14.9150244	-3.135576	0.88088698	-3.5595668	0.00037147	0.02316824
wild *M. balbisiana*	Ma07_g15570	36.4201782	-2.8503489	0.80180922	-3.5548966	0.00037813	0.02329889
wild *M. balbisiana*	Ma07_g24440	28.0301069	-1.9606128	0.55167478	-3.5539287	0.00037952	0.02329889
wild *M. balbisiana*	Ma08_g28840	88.2592557	2.14813869	0.60469466	3.55243534	0.00038168	0.02329889
wild *M. balbisiana*	Ma10_g18610	17.1384147	4.17228024	1.17692986	3.54505428	0.00039253	0.02379243
wild *M. balbisiana*	Ma02_g24260	8.67715574	5.1368321	1.45109479	3.53997004	0.00040017	0.0240859

Comparative RNA-seq analysis of resistant and susceptible banana genotypes reveals molecular mechanisms in response to *Banana bunchy top virus* (BBTV)

Darlon V. Lantican, Jen Daine L. Nocum, Anand Noel C. Manohar, Jay-Vee S. Mendoza, Roanne R. Gardoce, Grace C. Lachica, Lavernee S. Gueco and Fe M. Dela Cueva

Supplementary Table S2. List of differentially-expressed genes between mock- and BBTV-inoculated BBTV-resistant wild *M. balbisiana* and BBTV-susceptible Lakatan.

wild *M. balbisiana*	Ma04_g31130	25.4621123	-2.7516443	0.7778679	-3.5374185	0.00040406	0.02415094
wild *M. balbisiana*	Ma06_g33890	59.4444743	-1.630077	0.46127909	-3.5338194	0.0004096	0.02421702
wild *M. balbisiana*	Ma03_g28040	30.0517658	-3.7310934	1.05605393	-3.5330519	0.00041079	0.02421702
wild *M. balbisiana*	Ma08_g19890	216.276175	2.64710445	0.75039622	3.527609	0.00041933	0.02455226
wild *M. balbisiana*	Ma01_g17210	46.6562246	-2.1215357	0.60176113	-3.5255447	0.00042261	0.02457722
wild *M. balbisiana*	Ma05_g20170	15.9465502	-3.4393775	0.9767775	-3.5211473	0.00042968	0.02472269
wild *M. balbisiana*	Ma10_g08680	22.626819	-2.1543811	0.6119911	-3.5202818	0.00043109	0.02472269
wild *M. balbisiana*	Ma10_g18720	45.890082	-2.2213774	0.63131336	-3.5186605	0.00043373	0.02472269
wild *M. balbisiana*	Ma08_g12490	9.20670336	-3.9073249	1.1147741	-3.5050374	0.00045654	0.02561057
wild *M. balbisiana*	Ma04_g36890	10.1575451	-3.6423261	1.03942002	-3.5041908	0.000458	0.02561057
wild *M. balbisiana*	Ma08_g27190	9.47656649	3.61300413	1.03109301	3.50405259	0.00045823	0.02561057
wild *M. balbisiana*	Ma10_g19060	9.221637	4.10918609	1.17387479	3.50053185	0.00046433	0.02578384
wild *M. balbisiana*	Ma09_g01440	16.6455321	-2.8665139	0.82291954	-3.4833465	0.00049519	0.02716579
wild *M. balbisiana*	Ma08_g03140	28.2800716	-2.0547174	0.5899002	-3.483161	0.00049553	0.02716579
wild *M. balbisiana*	Ma06_g36550	8.37580833	-5.3218041	1.5296034	-3.4792052	0.0005029	0.02737737
wild *M. balbisiana*	Ma02_g15850	34.6370533	-1.9625684	0.56433077	-3.4776917	0.00050575	0.02737737
wild *M. balbisiana*	Ma03_g30520	26.1027124	2.31719818	0.66710844	3.47349554	0.00051373	0.02763524
wild *M. balbisiana*	Ma11_g22440	37.6668459	-2.0045639	0.57743229	-3.4715134	0.00051753	0.02766715
wild *M. balbisiana*	Ma06_g31950	21.8733673	-2.5529086	0.73600881	-3.4685843	0.00052321	0.02779787
wild *M. balbisiana*	Ma08_g25310	11.5042337	-3.100656	0.89592162	-3.4608563	0.00053846	0.02838944
wild *M. balbisiana*	Ma07_g19450	20.9468685	2.29874506	0.66445031	3.45961923	0.00054094	0.02838944
wild *M. balbisiana*	Ma08_g29650	10.037916	3.72264865	1.081164	3.4431859	0.0005749	0.02998908
wild *M. balbisiana*	Ma08_g30610	40.6898958	2.09678488	0.60936133	3.44095495	0.00057967	0.03005528
wild *M. balbisiana*	Ma07_g21110	62.3496702	1.94890253	0.56747418	3.43434574	0.00059399	0.0306134

Comparative RNA-seq analysis of resistant and susceptible banana genotypes reveals molecular mechanisms in response to *Banana bunchy top virus* (BBTV)

Darlon V. Lantican, Jen Daine L. Nocum, Anand Noel C. Manohar, Jay-Vee S. Mendoza, Roanne R. Gardoce, Grace C. Lachica, Lavernee S. Gueco and Fe M. Dela Cueva

Supplementary Table S2. List of differentially-expressed genes between mock- and BBTV-inoculated BBTV-resistant wild *M. balbisiana* and BBTV-susceptible Lakatan.

wild *M. balbisiana*	Ma03_g23180	15.1183053	2.36423517	0.68988988	3.42697473	0.00061035	0.03126933
wild *M. balbisiana*	Ma06_g27370	62.0079409	-1.9839679	0.58019636	-3.4194766	0.00062742	0.03195373
wild *M. balbisiana*	Ma07_g20780	20.9436239	-2.0325944	0.59470397	-3.4178255	0.00063124	0.03195909
wild *M. balbisiana*	Ma09_g07910	40.7608572	-2.2998732	0.67434054	-3.4105516	0.00064832	0.03249105
wild *M. balbisiana*	Ma11_g00670	7.81607723	-4.4244272	1.29743225	-3.4101412	0.00064929	0.03249105
wild *M. balbisiana*	Ma04_g06710	47.7859201	-1.5246264	0.44780205	-3.4046883	0.0006624	0.03295516
wild *M. balbisiana*	Ma06_g38760	181.77071	-1.5111908	0.44575121	-3.3902113	0.00069839	0.0345461
wild *M. balbisiana*	Ma06_g18390	21.9475451	2.14234514	0.63244012	3.38742765	0.00070551	0.03469916
wild *M. balbisiana*	Ma09_g06720	47.1050936	-2.637233	0.77952571	-3.383125	0.00071666	0.03504711
wild *M. balbisiana*	Ma11_g00040	26.8934197	2.24937216	0.66530563	3.38096064	0.00072233	0.03512477
wild *M. balbisiana*	Ma04_g37990	84.5336243	-1.3219264	0.39186619	-3.3734127	0.00074243	0.0358992
wild *M. balbisiana*	Ma07_g23960	23.4824172	-3.7392445	1.10952204	-3.3701399	0.0007513	0.03612537
wild *M. balbisiana*	Ma01_g15060	325.910359	2.11298843	0.62764219	3.36654938	0.00076115	0.03635634
wild *M. balbisiana*	Ma08_g05190	94.0023594	-2.0502006	0.60921431	-3.3653191	0.00076455	0.03635634
wild *M. balbisiana*	Ma10_g17310	115.30493	-1.5313779	0.45546508	-3.3622289	0.00077316	0.03656367
wild *M. balbisiana*	Ma04_g22180	15.1227785	2.53078068	0.75356644	3.35840419	0.00078394	0.03687083
wild *M. balbisiana*	Ma09_g05450	15.9820473	-2.8462548	0.85012007	-3.3480621	0.00081379	0.03806669
wild *M. balbisiana*	Ma10_g09050	98.6780892	2.58498492	0.77414824	3.33913427	0.0008404	0.03909901
wild *M. balbisiana*	Ma10_g26800	240.684886	-1.5744175	0.47207809	-3.3350786	0.00085275	0.0394133
wild *M. balbisiana*	Ma10_g15380	19.3412662	2.87198029	0.86144251	3.33391987	0.00085631	0.0394133
wild *M. balbisiana*	Ma03_g32450	76.0119869	-1.6796415	0.50453942	-3.3290589	0.0008714	0.03989435
wild *M. balbisiana*	Ma09_g20540	10.028864	-3.4125225	1.02820661	-3.3189074	0.0009037	0.04115439
wild *M. balbisiana*	Ma11_g03030	13.8697306	-4.7607204	1.43557254	-3.316252	0.00091234	0.04132878
wild *M. balbisiana*	Ma08_g28550	35.8217624	-1.7666634	0.53347625	-3.3116064	0.00092762	0.04180116

Comparative RNA-seq analysis of resistant and susceptible banana genotypes reveals molecular mechanisms in response to *Banana bunchy top virus* (BBTV)

Darlon V. Lantican, Jen Daine L. Nocum, Anand Noel C. Manohar, Jay-Vee S. Mendoza, Roanne R. Gardoce, Grace C. Lachica, Lavernee S. Gueco and Fe M. Dela Cueva

Supplementary Table S2. List of differentially-expressed genes between mock- and BBTV-inoculated BBTV-resistant wild *M. balbisiana* and BBTV-susceptible Lakatan.

wild *M. balbisiana*	Ma08_g31080	80.8623919	-1.4742615	0.44543079	-3.3097433	0.00093382	0.04186121
wild *M. balbisiana*	Ma08_g04840	13.9952898	-3.5366177	1.07106658	-3.3019588	0.00096012	0.04280018
wild *M. balbisiana*	Ma08_g04160	50.9357316	1.82699369	0.5535301	3.30062212	0.00096471	0.04280018
wild *M. balbisiana*	Ma11_g20540	22.9805053	2.17053138	0.65823014	3.29752656	0.0009754	0.04295514
wild *M. balbisiana*	Ma02_g08260	34.2270218	-2.0935462	0.63503757	-3.2967281	0.00097818	0.04295514
wild *M. balbisiana*	Ma03_g25870	54.1331429	1.88562209	0.57273726	3.29229859	0.00099372	0.04314616
wild *M. balbisiana*	Ma04_g09590	11.4321504	-3.1960364	0.97106592	-3.2912661	0.00099738	0.04314616
wild *M. balbisiana*	Ma09_g13780	16.5932587	-2.5642845	0.77913097	-3.2912112	0.00099757	0.04314616
wild *M. balbisiana*	Ma02_g12040	21.0030509	-3.3348595	1.01416605	-3.2882776	0.00100802	0.0433391
wild *M. balbisiana*	Ma06_g24210	59.2845592	1.61335006	0.49080648	3.28714092	0.0010121	0.0433391
wild *M. balbisiana*	Ma07_g28050	17.5536534	3.22452346	0.9827023	3.28128209	0.00103336	0.04403049
wild *M. balbisiana*	Ma01_g20450	22.2933823	-1.9210215	0.5857041	-3.2798499	0.00103862	0.04403661
wild *M. balbisiana*	Ma06_g05910	13.1229953	2.83748811	0.86850124	3.26710886	0.00108652	0.04584151
wild *M. balbisiana*	Ma02_g06390	15.5181552	2.76014965	0.84576054	3.26351199	0.00110041	0.04609015
wild *M. balbisiana*	Ma04_g08150	8.48890049	4.25945668	1.30545525	3.26281325	0.00110312	0.04609015
wild *M. balbisiana*	Ma06_g25800	175.439961	-2.640868	0.81141948	-3.2546273	0.00113541	0.04721013
wild *M. balbisiana*	Ma01_g17040	67.1428846	2.13272313	0.65642615	3.2489917	0.00115815	0.04774312
wild *M. balbisiana*	Ma09_g03420	36.3542753	2.75050503	0.84664716	3.24870282	0.00115933	0.04774312
wild *M. balbisiana*	Ma05_g25290	44.3695095	-1.7715224	0.54614628	-3.2436774	0.00117997	0.04836204
wild *M. balbisiana*	Ma04_g32970	20.583438	-2.4680058	0.76210569	-3.2384036	0.00120201	0.04861377
wild *M. balbisiana*	Ma11_g21720	13.7007067	2.50679482	0.77410892	3.23829727	0.00120245	0.04861377
wild *M. balbisiana*	Ma09_g14420	18.7222985	2.04331029	0.63101089	3.23815379	0.00120306	0.04861377
Lakatan	Ma07_g10990	102.256969	5.30208335	0.65925183	8.04257657	8.80E-16	8.71E-12
Lakatan	Ma07_g00170	395.892743	6.89999229	0.93055829	7.4148953	1.22E-13	6.03E-10

Supplementary Table S2. List of differentially-expressed genes between mock- and BBTV-inoculated BBTV-resistant wild *M. balbisiana* and BBTV-susceptible Lakatan.

Lakatan	Ma10_g11490	235.55852	7.87570646	1.22113142	6.44951588	1.12E-10	3.71E-07
Lakatan	Ma03_g03480	150.685808	7.2002445	1.14436641	6.29190478	3.14E-10	7.77E-07
Lakatan	Ma11_g01360	42.7809618	-4.9367858	0.81077981	-6.0889353	1.14E-09	2.25E-06
Lakatan	Ma02_g01300	47.0929747	-5.4062646	0.89558342	-6.0365841	1.57E-09	2.60E-06
Lakatan	Ma01_g16620	95.3458806	-4.1412768	0.70250493	-5.8950145	3.75E-09	5.30E-06
Lakatan	Ma08_g21950	34.5695299	-6.894889	1.17995107	-5.8433686	5.12E-09	6.33E-06
Lakatan	Ma08_g10330	112.058447	3.05053604	0.5592708	5.45448829	4.91E-08	5.41E-05
Lakatan	Ma01_g22550	214.484552	-5.218621	0.97246984	-5.3663577	8.03E-08	7.96E-05
Lakatan	Ma03_g32970	81.0751161	-3.3752936	0.63252635	-5.3362103	9.49E-08	8.55E-05
Lakatan	Ma07_g12370	34.9481353	5.04016416	0.9539667	5.28337539	1.27E-07	0.00010469
Lakatan	Ma09_g08600	45.4665777	4.07478123	0.77623894	5.24939038	1.53E-07	0.00011628
Lakatan	Ma01_g21040	52.2448462	-5.7163384	1.09898296	-5.2014804	1.98E-07	0.00013989
Lakatan	Ma09_g25280	73.2147746	3.19489558	0.61892041	5.16204596	2.44E-07	0.00016131
Lakatan	Ma00_g00510	74.9387211	5.76194099	1.14574289	5.029	4.93E-07	0.00030526
Lakatan	Ma06_g33250	62.4409956	-4.6371312	0.92644141	-5.0053151	5.58E-07	0.00032498
Lakatan	Ma06_g36640	35.1258952	4.46119053	0.89501931	4.98446288	6.21E-07	0.00033583
Lakatan	Ma10_g20080	153.130761	-4.4419398	0.8924049	-4.9774937	6.44E-07	0.00033583
Lakatan	Ma06_g18390	46.7780566	3.78122568	0.77474069	4.88063392	1.06E-06	0.00051139
Lakatan	Ma05_g15310	83.223802	-3.6618186	0.75103091	-4.8757228	1.08E-06	0.00051139
Lakatan	Ma01_g01670	199.589805	-3.2584962	0.67032538	-4.8610664	1.17E-06	0.00052572
Lakatan	Ma04_g16680	147.102042	-3.3838376	0.7039842	-4.8066955	1.53E-06	0.00066088
Lakatan	Ma05_g12140	23.7764283	5.08685159	1.06754565	4.76499677	1.89E-06	0.00075844
Lakatan	Ma06_g12410	25.9513459	-4.5498359	0.95538845	-4.762289	1.91E-06	0.00075844
Lakatan	Ma02_g00510	32.3812996	3.24491236	0.68790071	4.71712316	2.39E-06	0.00091136

Comparative RNA-seq analysis of resistant and susceptible banana genotypes reveals molecular mechanisms in response to *Banana bunchy top virus* (BBTV)

Darlon V. Lantican, Jen Daine L. Nocum, Anand Noel C. Manohar, Jay-Vee S. Mendoza, Roanne R. Gardoce, Grace C. Lachica, Lavernee S. Gueco and Fe M. Dela Cueva

Supplementary Table S2. List of differentially-expressed genes between mock- and BBTV-inoculated BBTV-resistant wild *M. balbisiana* and BBTV-susceptible Lakatan.

Lakatan	Ma08_g07100	62.5904816	3.28477174	0.70314808	4.671522	2.99E-06	0.00109691
Lakatan	Ma10_g15170	219.903607	-3.3953304	0.72968147	-4.6531679	3.27E-06	0.00115643
Lakatan	Ma02_g17130	252.76372	-2.4896422	0.54047639	-4.6063848	4.10E-06	0.00139958
Lakatan	Ma08_g15460	36.7820919	-4.4209429	0.96740942	-4.5698779	4.88E-06	0.00160532
Lakatan	Ma11_g06590	111.47152	2.42889173	0.53220883	4.56379452	5.02E-06	0.00160532
Lakatan	Ma09_g03420	33.612383	3.90610484	0.86244441	4.52910912	5.92E-06	0.00183363
Lakatan	Ma10_g21920	62.2412982	-3.3207243	0.73556049	-4.5145496	6.35E-06	0.0019047
Lakatan	Ma11_g14200	80.8291774	-3.0735804	0.68574606	-4.4820971	7.39E-06	0.00215348
Lakatan	Ma02_g24260	26.4744137	5.21925161	1.1703028	4.45974463	8.21E-06	0.00227165
Lakatan	Ma09_g22570	128.832703	2.80156984	0.62899951	4.45400955	8.43E-06	0.00227165
Lakatan	Ma08_g10850	63.6720501	-3.0946675	0.69532394	-4.4506846	8.56E-06	0.00227165
Lakatan	Ma03_g05910	96.5238209	-4.0566985	0.91226507	-4.4468419	8.71E-06	0.00227165
Lakatan	Ma09_g30010	57.5119985	2.81438251	0.63478512	4.43359876	9.27E-06	0.00235389
Lakatan	Ma02_g18000	134.937842	-3.2698645	0.74339398	-4.398562	1.09E-05	0.00269865
Lakatan	Ma11_g07100	246.054449	-2.6578086	0.60649695	-4.3822291	1.17E-05	0.00283822
Lakatan	Ma02_g14600	21.3344341	3.76668958	0.86457274	4.35670637	1.32E-05	0.00311412
Lakatan	Ma03_g29410	244.242434	-4.7641815	1.09660304	-4.3444906	1.40E-05	0.00321598
Lakatan	Ma04_g31960	17.5639134	3.78489723	0.87245555	4.33821212	1.44E-05	0.003234
Lakatan	Ma11_g14880	19.1334581	3.95302175	0.91385	4.32567898	1.52E-05	0.00334741
Lakatan	Ma10_g19080	255.318051	-2.7359012	0.63407955	-4.3147602	1.60E-05	0.00344075
Lakatan	Ma06_g29050	301.297634	-3.1420661	0.72989671	-4.3048092	1.67E-05	0.00352253
Lakatan	Ma03_g31670	93.5723511	-2.4862801	0.57979841	-4.2881802	1.80E-05	0.00371099
Lakatan	Ma01_g09220	71.1631258	2.4002617	0.56028536	4.28399859	1.84E-05	0.00371099
Lakatan	Ma03_g14330	26.4147918	-2.6622784	0.62222895	-4.2786155	1.88E-05	0.00372583

Comparative RNA-seq analysis of resistant and susceptible banana genotypes reveals molecular mechanisms in response to *Banana bunchy top virus* (BBTV)

Darlon V. Lantican, Jen Daine L. Nocum, Anand Noel C. Manohar, Jay-Vee S. Mendoza, Roanne R. Gardoce, Grace C. Lachica, Lavernee S. Gueco and Fe M. Dela Cueva

Supplementary Table S2. List of differentially-expressed genes between mock- and BBTV-inoculated BBTV-resistant wild *M. balbisiana* and BBTV-susceptible Lakatan.

Lakatan	Ma06_g31170	72.282478	3.50423785	0.82274758	4.25918949	2.05E-05	0.00398512
Lakatan	Ma08_g23180	868.355888	3.39680301	0.80872781	4.20018079	2.67E-05	0.00508067
Lakatan	Ma06_g30390	820.685634	3.44647391	0.82532181	4.17591523	2.97E-05	0.00554718
Lakatan	Ma09_g06560	105.176148	-3.1173481	0.7499153	-4.1569336	3.23E-05	0.00591696
Lakatan	Ma01_g15210	45.530882	2.09440819	0.50460091	4.15062312	3.32E-05	0.0059719
Lakatan	Ma06_g35530	207.364919	3.74347115	0.90570119	4.13322978	3.58E-05	0.00622974
Lakatan	Ma06_g32100	88.5016661	2.02812544	0.49074601	4.13273953	3.58E-05	0.00622974
Lakatan	Ma08_g21090	46.8055026	-2.7428978	0.66781199	-4.1072904	4.00E-05	0.00683732
Lakatan	Ma01_g04190	26.4472928	-2.7399732	0.66776811	-4.1031806	4.08E-05	0.006842
Lakatan	Ma04_g01330	260.231555	-2.257402	0.55362292	-4.0775082	4.55E-05	0.0075155
Lakatan	Ma03_g10270	61.1863791	3.07187215	0.75667145	4.05971727	4.91E-05	0.00797874
Lakatan	Ma10_g11130	140.752652	-3.0921129	0.76578669	-4.0378253	5.39E-05	0.00861966
Lakatan	Ma06_g16330	27.5383845	-3.4342823	0.85627553	-4.0107211	6.05E-05	0.00942232
Lakatan	Ma03_g21600	32.7767255	6.1085455	1.52355876	4.00939278	6.09E-05	0.00942232
Lakatan	Ma04_g14870	147.552591	-2.0456123	0.51117235	-4.0018054	6.29E-05	0.00950189
Lakatan	Ma08_g24690	54.5165044	2.73055306	0.68265481	3.99990304	6.34E-05	0.00950189
Lakatan	Ma05_g25290	133.516671	-2.3539228	0.58898568	-3.9965704	6.43E-05	0.00950189
Lakatan	Ma10_g05590	258.475504	-3.1709081	0.79797388	-3.9736991	7.08E-05	0.01030879
Lakatan	Ma06_g00750	59.8126234	-2.8877816	0.72841482	-3.964474	7.36E-05	0.01056037
Lakatan	Ma11_g20430	143.196031	1.6189345	0.4089984	3.95829058	7.55E-05	0.01066893
Lakatan	Ma07_g13740	205.539329	-3.1490096	0.79616795	-3.9552077	7.65E-05	0.01066893
Lakatan	Ma01_g07220	111.795089	-2.6002839	0.66303823	-3.9217707	8.79E-05	0.01209366
Lakatan	Ma00_g01690	115.120741	2.30603142	0.59003563	3.9082918	9.30E-05	0.01256386
Lakatan	Ma10_g08680	51.4919519	-2.300931	0.58917384	-3.9053517	9.41E-05	0.01256386

Supplementary Table S2. List of differentially-expressed genes between mock- and BBTV-inoculated BBTV-resistant wild *M. balbisiana* and BBTV-susceptible Lakatan.

Lakatan	Ma10_g19060	14.7889704	4.29390994	1.10023908	3.90270625	9.51E-05	0.01256386
Lakatan	Ma05_g21470	79.9908685	-2.4685832	0.63586123	-3.8822673	0.00010349	0.01344449
Lakatan	Ma10_g14160	147.710028	-3.4675062	0.89371316	-3.8798871	0.0001045	0.01344449
Lakatan	Ma04_g37140	144.853232	1.84767344	0.47706909	3.87296829	0.00010752	0.01365476
Lakatan	Ma04_g37990	212.544696	-2.0815241	0.54101296	-3.8474571	0.00011935	0.0149172
Lakatan	mito3_g00190	4324.51947	-3.0435493	0.7917052	-3.8442962	0.0001209	0.0149172
Lakatan	Ma03_g17480	139.005528	-3.1380071	0.81712784	-3.840289	0.00012289	0.0149172
Lakatan	Ma06_g36160	71.8041679	-2.9452638	0.76717385	-3.8391087	0.00012348	0.0149172
Lakatan	Ma05_g08440	16.2015383	4.9220099	1.2860257	3.82730291	0.00012956	0.0153685
Lakatan	Ma06_g23640	108.088103	2.74544842	0.71760439	3.82585231	0.00013032	0.0153685
Lakatan	Ma11_g12630	53.6258245	2.55505828	0.66844609	3.82238497	0.00013217	0.01540291
Lakatan	Ma03_g05660	39.4140545	4.45631175	1.16722335	3.81787406	0.00013461	0.0155048
Lakatan	Ma06_g18280	134.034927	-1.7331933	0.45520931	-3.8074645	0.0001404	0.0159861
Lakatan	Ma09_g08110	149.897963	2.85167497	0.75139875	3.79515531	0.00014755	0.01650746
Lakatan	Ma06_g26470	74.732237	-2.9863659	0.7871532	-3.7938815	0.00014831	0.01650746
Lakatan	Ma09_g04960	102.072693	-2.447174	0.64656272	-3.784898	0.00015377	0.01690872
Lakatan	Ma07_g09610	442.925616	2.90005091	0.76700943	3.78098471	0.00015621	0.01690872
Lakatan	Ma08_g10940	89.840984	-3.1152796	0.82438407	-3.7789177	0.00015751	0.01690872
Lakatan	Ma06_g30910	93.9324146	-3.2066659	0.84900326	-3.7769771	0.00015874	0.01690872
Lakatan	Ma04_g15280	16.1952172	-4.3243128	1.14945649	-3.76205	0.00016853	0.01775981
Lakatan	Ma07_g15780	21.3154844	-5.5405674	1.48639232	-3.7275269	0.00019337	0.01985189
Lakatan	Ma11_g13780	144.316485	2.36212275	0.6339	3.72633341	0.00019429	0.01985189
Lakatan	Ma06_g25800	155.48361	-3.1099949	0.83477097	-3.7255666	0.00019488	0.01985189
Lakatan	Ma06_g27370	164.099516	-2.2425841	0.6027283	-3.7207214	0.00019865	0.01985189

Supplementary Table S2. List of differentially-expressed genes between mock- and BBTV-inoculated BBTV-resistant wild *M. balbisiana* and BBTV-susceptible Lakatan.

Lakatan	Ma04_g12630	30.8108896	3.00359343	0.8072914	3.72058145	0.00019876	0.01985189
Lakatan	Ma10_g20950	321.94642	3.54902781	0.95488793	3.71669564	0.00020185	0.01985189
Lakatan	Ma05_g04870	33.5844949	2.38887495	0.64286305	3.71599354	0.00020241	0.01985189
Lakatan	Ma08_g06150	21.5297701	3.8962742	1.0505391	3.70883311	0.00020822	0.02016116
Lakatan	Ma08_g22930	37.3683381	-2.2159853	0.5977648	-3.707119	0.00020963	0.02016116
Lakatan	Ma03_g29390	27.3235739	3.26881245	0.88277027	3.70290273	0.00021315	0.02030222
Lakatan	Ma06_g09580	128.31664	1.43842216	0.38984897	3.68969081	0.00022453	0.0211825
Lakatan	Ma06_g32730	20.5737377	2.93390157	0.79728293	3.67987505	0.00023335	0.02180706
Lakatan	Ma06_g27170	16.5523282	-5.1760279	1.41477361	-3.6585556	0.00025364	0.02328941
Lakatan	Ma06_g00390	34.5350919	-2.2662746	0.61949165	-3.6582812	0.00025391	0.02328941
Lakatan	Ma11_g09120	38.6865311	2.60964995	0.71629059	3.64328388	0.00026918	0.02446343
Lakatan	Ma07_g07360	23.3449646	2.53686736	0.69736333	3.63779865	0.00027498	0.02454183
Lakatan	Ma08_g22590	25.6638948	-3.0522339	0.83903776	-3.6377789	0.000275	0.02454183
Lakatan	Ma06_g01470	50.7643043	1.99600983	0.55026318	3.62737304	0.00028632	0.02532394
Lakatan	Ma03_g14240	294.747374	1.56398331	0.4318314	3.62174521	0.00029262	0.02546559
Lakatan	Ma06_g16250	17.8058158	3.88499688	1.07280161	3.62135631	0.00029306	0.02546559
Lakatan	Ma03_g02600	85.5276769	-2.0608352	0.5698688	-3.6163327	0.00029881	0.02573892
Lakatan	Ma02_g05270	196.422946	3.67977896	1.01971126	3.60864796	0.0003078	0.0262848
Lakatan	Ma10_g26330	45.1557221	4.18511818	1.16228281	3.60077439	0.00031727	0.02686226
Lakatan	Ma11_g08690	17.5192369	-5.1898113	1.44395527	-3.5941635	0.00032544	0.02732003
Lakatan	Ma07_g21640	16.5259027	2.60924774	0.72750733	3.58655872	0.00033507	0.02789252
Lakatan	Ma00_g02720	556.855253	-1.5602374	0.43551395	-3.5825199	0.0003403	0.02809141
Lakatan	Ma10_g20770	13.8379083	3.53500786	0.99175515	3.56439578	0.0003647	0.02985679
Lakatan	Ma03_g18330	1037.12345	-2.3614096	0.66346565	-3.559204	0.00037198	0.03004634

Supplementary Table S2. List of differentially-expressed genes between mock- and BBTV-inoculated BBTV-resistant wild *M. balbisiana* and BBTV-susceptible Lakatan.

Lakatan	Ma03_g23150	96.0507547	2.34660882	0.65945043	3.55843094	0.00037308	0.03004634
Lakatan	Ma02_g21520	38.6121347	-4.3110497	1.21448235	-3.5497014	0.00038567	0.03080992
Lakatan	Ma08_g27750	22.9390021	2.69830482	0.76296292	3.53661332	0.00040529	0.03211863
Lakatan	Ma02_g14310	20.0126788	-3.1945888	0.90434073	-3.5325057	0.00041164	0.03232746
Lakatan	Ma11_g15590	34.3525048	2.83810911	0.80383651	3.53070441	0.00041445	0.03232746
Lakatan	Ma10_g13420	126.717543	1.38743229	0.39410137	3.52049599	0.00043074	0.03333528
Lakatan	Ma03_g08170	38.8310624	3.06668924	0.87185698	3.51742235	0.00043576	0.0334623
Lakatan	Ma02_g24860	194.054274	-2.4745564	0.70580261	-3.5060177	0.00045486	0.03466071
Lakatan	Ma06_g31610	98.6856995	1.66150085	0.47693489	3.48370579	0.00049452	0.03739498
Lakatan	Ma01_g14940	574.532748	-2.2189807	0.63758802	-3.4802736	0.0005009	0.03759042
Lakatan	Ma03_g25870	62.4296574	2.2877144	0.6593097	3.46986309	0.00052072	0.03878413
Lakatan	Ma07_g11510	25.7596584	2.89410445	0.83462706	3.46754206	0.00052524	0.03879018
Lakatan	Ma03_g20940	90.2194059	-2.3197739	0.66958098	-3.4645159	0.00053119	0.03879018
Lakatan	Ma03_g05310	41.0383821	-1.9713776	0.56913311	-3.4638252	0.00053255	0.03879018
Lakatan	Ma09_g10410	42.638606	-3.2034425	0.92620317	-3.4586823	0.00054282	0.03924979
Lakatan	Ma05_g03580	56.0533071	-4.2301399	1.22613281	-3.4499851	0.00056062	0.03970835
Lakatan	Ma11_g03670	36.1170675	2.3680479	0.68656038	3.44914735	0.00056236	0.03970835
Lakatan	Ma09_g03120	21.2833652	-2.6151874	0.75860325	-3.4473717	0.00056607	0.03970835
Lakatan	Ma02_g07680	17.948552	2.86980136	0.83257068	3.44691619	0.00056702	0.03970835
Lakatan	Ma09_g06790	64.895146	2.55120686	0.7404251	3.44559747	0.0005698	0.03970835
Lakatan	Ma08_g21820	396.425663	-1.884064	0.54706006	-3.4439802	0.00057322	0.03970835
Lakatan	Ma10_g19400	15.7573904	4.70231043	1.36715906	3.43947574	0.00058284	0.04009467
Lakatan	Ma06_g25920	45.5421148	1.93187393	0.56260185	3.43382079	0.00059514	0.04048733
Lakatan	Ma10_g13860	46.5011843	2.08904279	0.6085006	3.43309899	0.00059672	0.04048733

Supplementary Table S2. List of differentially-expressed genes between mock- and BBTV-inoculated BBTV-resistant wild *M. balbisiana* and BBTV-susceptible Lakatan.

Lakatan	Ma03_g01310	189.937436	-1.6001193	0.46690907	-3.4270469	0.00061018	0.0411189
Lakatan	Ma08_g05970	15.6089614	2.91222461	0.85242676	3.41639277	0.00063457	0.04247308
Lakatan	Ma03_g30490	20.6974277	2.5581271	0.75086837	3.40689154	0.00065707	0.04349677
Lakatan	Ma02_g24950	14.4704094	-2.9008541	0.85176626	-3.4056926	0.00065996	0.04349677
Lakatan	Ma07_g20820	479.957647	-2.717043	0.79809148	-3.4044255	0.00066303	0.04349677
Lakatan	Ma05_g00150	16.0274692	-4.0785452	1.20621605	-3.3812726	0.00072151	0.04702152
Lakatan	Ma04_g21660	132.442071	-2.7326522	0.80884425	-3.3784653	0.00072892	0.04719376
Lakatan	Ma06_g14630	31.8187809	-1.8219677	0.5400487	-3.3737099	0.00074162	0.04770477
Lakatan	Ma04_g33050	161.150581	-1.8201995	0.54015897	-3.3697479	0.00075237	0.04808372
Lakatan	Ma06_g22120	21.5102841	4.45667834	1.32426578	3.36539568	0.00076434	0.04828559
Lakatan	mito11_g00040	6941.2418	-1.3077083	0.38861398	-3.3650574	0.00076528	0.04828559
Lakatan	Ma11_g02330	43.585705	-3.1687216	0.94290368	-3.3605995	0.00077774	0.04876104
Lakatan	Ma07_g25830	32.9271186	-3.0254152	0.90124886	-3.3569143	0.00078818	0.04910481
Lakatan	Ma04_g39130	81.2862477	2.40178643	0.71661139	3.35158841	0.00080349	0.04960405
Lakatan	Ma09_g00790	52.2579742	-3.0356552	0.90598828	-3.3506561	0.0008062	0.04960405
