# Supplementary material for: Comparative RNA-seq analysis of resistant and susceptible banana genotypes reveals molecular mechanisms in response to banana bunchy top virus (BBTV) infection
Source: Sci Rep. 2023 Oct 31;13:18719. doi: 10.1038/s41598-023-45937-z (PMC10618458; doi:10.1038/s41598-023-45937-z)
Supplement: Supplementary file 3 — Supplementary Table S3. [file 41598_2023_45937_MOESM3_ESM.pdf]

Comparative RNA-seq analysis of resistant and susceptible banana genotypes reveals molecular mechanisms in response to *Banana bunchy top virus* (BBTV) infection

Darlon V. Lantican, Jen Daine L. Nocum, Anand Noel C. Manohar, Jay-Vee S. Mendoza, Roanne R. Gardoce, Grace C. Lachica, Lavernee S. Gueco and Fe M. Dela Cueva

Supplementary Table S3. List of enriched (padj < 0.075) gene ontology terms in response of *wild M. balbisiana* and Lakatan to BBTV inoculation with mock-inoculated samples as baseline control.

Genotype	GOType	ID	Description	GeneRatio	BgRatio	pvalue	p.adjust	qvalue	Count
wild M. balbisiana	Biological Process	GO:0005985	sucrose metabolic process	4/184	24/34320	7.81E-06	0.00115238	0.00095398	4
wild M. balbisiana	Biological Process	GO:0007623	circadian rhythm	5/184	87/34320	0.00010859	0.00678848	0.00561972	5
wild M. balbisiana	Biological Process	GO:0042752	regulation of circadian rhythm	4/184	46/34320	0.00010943	0.00678848	0.00561972	4
wild M. balbisiana	Biological Process	GO:0071456	cellular response to hypoxia	5/184	116/34320	0.00041596	0.01873718	0.01551124	5
wild M. balbisiana	Biological Process	GO:0009640	photomorphogenesis	4/184	66/34320	0.00044461	0.01873718	0.01551124	4
wild M. balbisiana	Biological Process	GO:0009704	de-etiolation	2/184	10/34320	0.00125058	0.03689217	0.03054053	2
wild M. balbisiana	Biological Process	GO:0072488	ammonium transmembrane transport	2/184	12/34320	0.00182129	0.04218209	0.03491969	2
wild M. balbisiana	Biological Process	GO:0015977	carbon fixation	2/184	13/34320	0.00214485	0.04218209	0.03491969	2
wild M. balbisiana	Biological Process	GO:0010597	green leaf volatile biosynthetic process	3/184	49/34320	0.0023301	0.04296127	0.03556473	3
wild M. balbisiana	Biological Process	GO:0009813	flavonoid biosynthetic process	3/184	51/34320	0.0026132	0.04534676	0.03753951	3

Comparative RNA-seq analysis of resistant and susceptible banana genotypes reveals molecular mechanisms in response to *Banana bunchy top virus* (BBTV) infection

Darlon V. Lantican, Jen Daine L. Nocum, Anand Noel C. Manohar, Jay-Vee S. Mendoza, Roanne R. Gardoce, Grace C. Lachica, Lavernee S. Gueco and Fe M. Dela Cueva

Supplementary Table S3. List of enriched (padj < 0.075) gene ontology terms in response of *wild M. balbisiana* and Lakatan to BBTV inoculation with mock-inoculated samples as baseline control.

wild M. balbisiana	Molecular Function	GO:0016157	sucrose synthase activity	4/184	14/34320	7.68E-07	0.00022642	0.00018744	4
wild M. balbisiana	Molecular Function	GO:0008237	metallopeptidase activity	3/184	30/34320	0.00055326	0.02040163	0.01688913	3
wild M. balbisiana	Molecular Function	GO:0015301	anion:anion antiporter activity	2/184	12/34320	0.00182129	0.04218209	0.03491969	2
wild M. balbisiana	Molecular Function	GO:0008519	ammonium transmembrane transporter activity	2/184	13/34320	0.00214485	0.04218209	0.03491969	2
wild M. balbisiana	Molecular Function	GO:0004311	farnesyltranstransferase activity	2/184	15/34320	0.00286702	0.04698726	0.03889757	2
wild M. balbisiana	Molecular Function	GO:0016620	oxidoreductase activity, acting on the aldehyde or oxo group of donors, NAD or NADP as acceptor	3/184	54/34320	0.00307616	0.04776139	0.03953842	3
Lakatan	Biological Process	GO:0042752	regulation of circadian rhythm	6/142	46/34320	3.69E-08	9.84E-06	7.68E-06	6
Lakatan	Biological Process	GO:0007623	circadian rhythm	7/142	87/34320	7.93E-08	1.06E-05	8.26E-06	7
Lakatan	Biological Process	GO:0048511	rhythmic process	5/142	62/34320	6.05E-06	0.00040366	0.0003151	5
Lakatan	Biological Process	GO:0009640	photomorphogenesis	5/142	66/34320	8.24E-06	0.0004401	0.00034355	5

Comparative RNA-seq analysis of resistant and susceptible banana genotypes reveals molecular mechanisms in response to *Banana bunchy top virus* (BBTV) infection

Darlon V. Lantican, Jen Daine L. Nocum, Anand Noel C. Manohar, Jay-Vee S. Mendoza, Roanne R. Gardoce, Grace C. Lachica, Lavernee S. Gueco and Fe M. Dela Cueva

Supplementary Table S3. List of enriched (padj < 0.075) gene ontology terms in response of *wild M. balbisiana* and Lakatan to BBTV inoculation with mock-inoculated samples as baseline control.

Lakatan	Biological Process	GO:0009637	response to blue light	4/142	34/34320	1.18E-05	0.00052635	0.00041087	4
Lakatan	Biological Process	GO:0006457	protein folding	6/142	201/34320	0.00019791	0.00754897	0.00589275	6
Lakatan	Biological Process	GO:0009615	response to virus	3/142	36/34320	0.00044797	0.014662	0.0114452	3
Lakatan	Biological Process	GO:0009408	response to heat	5/142	156/34320	0.00049422	0.014662	0.0114452	5
Lakatan	Biological Process	GO:0042026	protein refolding	3/142	44/34320	0.0008111	0.01714831	0.01338603	3
Lakatan	Biological Process	GO:0051085	chaperone cofactor-dependent protein refolding	3/142	47/34320	0.0009841	0.01714831	0.01338603	3
Lakatan	Biological Process	GO:0006354	DNA-templated transcription, elongation	2/142	12/34320	0.00109184	0.01714831	0.01338603	2
Lakatan	Biological Process	GO:0010187	negative regulation of seed germination	2/142	13/34320	0.00128686	0.0190884	0.01490047	2
Lakatan	Biological Process	GO:0009649	entrainment of circadian clock	3/142	54/34320	0.00147392	0.02071243	0.01616819	3
Lakatan	Biological Process	GO:0009266	response to temperature stimulus	2/142	15/34320	0.00172293	0.02297043	0.01793079	2

Comparative RNA-seq analysis of resistant and susceptible banana genotypes reveals molecular mechanisms in response to *Banana bunchy top virus* (BBTV) infection

Darlon V. Lantican, Jen Daine L. Nocum, Anand Noel C. Manohar, Jay-Vee S. Mendoza, Roanne R. Gardoce, Grace C. Lachica, Lavernee S. Gueco and Fe M. Dela Cueva

Supplementary Table S3. List of enriched (padj < 0.075) gene ontology terms in response of *wild M. balbisiana* and Lakatan to BBTV inoculation with mock-inoculated samples as baseline control.

Lakatan	Biological Process	GO:0045892	negative regulation of transcription, DNA-templated	6/142	307/34320	0.00180666	0.02297043	0.01793079	6
Lakatan	Biological Process	GO:0006396	RNA processing	4/142	129/34320	0.00207308	0.02406572	0.01878578	4
Lakatan	Biological Process	GO:2000028	regulation of photoperiodism, flowering	3/142	76/34320	0.00390993	0.041758	0.03259643	3
Lakatan	Biological Process	GO:0034620	cellular response to unfolded protein	2/142	24/34320	0.00441979	0.04370685	0.0341177	2
Lakatan	Biological Process	GO:0048586	regulation of long-day photoperiodism, flowering	2/142	25/34320	0.00479115	0.04507847	0.0351884	2
Lakatan	Biological Process	GO:0009058	biosynthetic process	4/142	164/34320	0.00489616	0.04507847	0.0351884	4
Lakatan	Molecular Function	GO:0051082	unfolded protein binding	7/142	162/34320	5.34E-06	0.00040366	0.0003151	7
Lakatan	Molecular Function	GO:0031072	heat shock protein binding	3/142	48/34320	0.00104654	0.01714831	0.01338603	3
Lakatan	Molecular Function	GO:0015301	anion:anion antiporter activity	2/142	12/34320	0.00109184	0.01714831	0.01338603	2
Lakatan	Molecular Function	GO:0046914	transition metal ion binding	2/142	12/34320	0.00109184	0.01714831	0.01338603	2

Comparative RNA-seq analysis of resistant and susceptible banana genotypes reveals molecular mechanisms in response to *Banana bunchy top virus* (BBTV) infection

Darlon V. Lantican, Jen Daine L. Nocum, Anand Noel C. Manohar, Jay-Vee S. Mendoza, Roanne R. Gardoce, Grace C. Lachica, Lavernee S. Gueco and Fe M. Dela Cueva

Supplementary Table S3. List of enriched (padj < 0.075) gene ontology terms in response of *wild M. balbisiana* and Lakatan to BBTV inoculation with mock-inoculated samples as baseline control.

Lakatan	Molecular Function	GO:0033926	glycopeptide alpha-N-acetylgalactosaminidase activity	2/142	16/34320	0.00196373	0.0238326	0.01860381	2
Lakatan	Molecular Function	GO:0070063	RNA polymerase binding	2/142	17/34320	0.00221954	0.02469239	0.01927496	2
Lakatan	Molecular Function	GO:0051787	misfolded protein binding	2/142	24/34320	0.00441979	0.04370685	0.0341177	2
Lakatan	Cellular Component	GO:0009570	chloroplast stroma	6/142	271/34320	0.00095809	0.01714831	0.01338603	6
