# Supplementary material for: Comparative RNA-seq analysis of resistant and susceptible banana genotypes reveals molecular mechanisms in response to banana bunchy top virus (BBTV) infection
Source: Sci Rep. 2023 Oct 31;13:18719. doi: 10.1038/s41598-023-45937-z (PMC10618458; doi:10.1038/s41598-023-45937-z)
Supplement: Supplementary file 4 — Supplementary Table S4. [file 41598_2023_45937_MOESM4_ESM.pdf]

Comparative RNA-seq analysis of resistant and susceptible banana genotypes reveals molecular mechanisms in response to *Banana bunchy top virus* (BBTV) infection

Darlon V. Lantican, Jen Daine L. Nocum, Anand Noel C. Manohar, Jay-Vee S. Mendoza, Roanne R. Gardoce, Grace C. Lachica, Lavernee S. Gueco and Fe M. Dela Cueva

Supplementary Table S4. Validation of differentially expressed genes with RT-qPCR and concordance to RNA-seq analysis.

GENE ID	*GENE FUNCTION*	Forward Sequence	Reverse Sequence	RNA-Seq fold change	qPCR validation fold change	Concordant? (yes/no)
Ma07_g10990	*Adagio-like protein*	TCGAGAACGCCACCGGATAC	AGCGGAGTGCCATCCTTCCTA	5.302083346	30.31	yes
Ma06_g31610	*Probable zinc metalloprotease EGY1*	ACGAGGACCTGAACTCGGA	TCCAAGCAGTTCCAGAAGCT	1.661500848	4.52	yes
Ma10_g05760	*Nematode resistance protein-like HSPRO2*	AGACTTCTGGCAGACCGAT	AACGCTTGTCAGTGCCACT	-2.854365873	2.107297177	no
Ma07_g23140	*protein detoxification*	CTGAGGTTGCCATAGATGC	CCTGCTGCTGACAAGAAAC	-2.973160419	0.715247784	yes
Ma07_g26720	*leucine rich repeat protein kinase VI*	GCTTGAAGCTGCTTGTGAGG	TCGGTAGATGTGATGGTGGTCG	-2.40301635	4.729922787	no
Ma06_g30390	*light regulated protein*	ATCGCGCCCAGGTCATCCAA	ACGCCTCTCCACCAACTACA	3.446473912	3.628448845	yes
Ma03_g28040	*chitinase*	GCAGACCTCTCATGAAACC	CTATAGCTTTCCCAGCTTGG	-3.731093357	0.876714068	yes
Ma08_g29650	*Glucuronoxylan 4-O-methyltransferase*	CCATCGTCCACTACACCACC	GCCGAAGACCAGGAAGTTGC	3.722648648	14.28070438	yes
Ma11_g13780	*BTB domain-containing protein*	CATCCTCCAATCCCTCCAAG	CTCAGCTCCGTGTATACTTTC	2.36212275	4.16334547	yes
Ma02_g14310	*EDR2_C domain-containing protein*	CGTCCTCAGCATGATTTC	CAGCTTCAGCCTGTCAAG	-3.194588833	1.791539176	no
Ma08_g06150	*alkaline/neutral invertase*	CTAGTCACTGATGGCTCTTGC	CAGTCTGTTATTGATAGCACGC	3.896274205	3.115384629	yes
Ma11_g20430	*RuBisCO*	CAATGCTGTCGGTGTTAC	GCACGAGCAATAGTTACC	1.618934505	5.879492288	yes
Ma11_g09120	*Eukaryotic translation initiation factor 5A-5*	GGTACATCGTCATCAAGAACAG	GAAGATGGCACAATATCCTCAAG	2.609649947	2.190776629	yes
Ma06_g32730	*J domain-containing protein*	GAAACCCAATGAGAACAGCCAAG	CGATGAAGTCCAGCAATCTTG	2.933901567	2.024284925	yes
Ma03_g29390	*HATPase C domain-containing protein*	CCTGGAGATACGGATCAAGC	GAACTTTGACGTACCACTCTG	3.268812451	6.549655028	yes
Ma03_g29391	*APETALA-like 2*	CAATGGAAGGGAGGCAGTC	GGACTATGAACATCAGGTTG	-1.884064013	4.071357462	no
internal control gene	*L2*	AGGGTTCATAGCCACACCAC	CCGAACTGAGAAGCCCCTAC			

Comparative RNA-seq analysis of resistant and susceptible banana genotypes reveals molecular mechanisms in response to *Banana bunchy top virus* (BBTV) infection

Darlon V. Lantican, Jen Daine L. Nocum, Anand Noel C. Manohar, Jay-Vee S. Mendoza, Roanne R. Gardoce, Grace C. Lachica, Lavernee S. Gueco and Fe M. Dela Cueva

Supplementary Table S4. Validation of differentially expressed genes with RT-qPCR and concordance to RNA-seq analysis.
